# Characterisation of Adobe and Mud–Straw for the Restoration and Rehabilitation of Persian Historical Adobe Buildings

**DOI:** 10.3390/ma17081764

**Published:** 2024-04-11

**Authors:** Bina Hejazi, Corinna Luz, Friedrich Grüner, Jürgen Frick, Harald Garrecht

**Affiliations:** Materials Testing Institute (MPA), University of Stuttgart, 70569 Stuttgart, Germany; corinna.luz@mpa.uni-stuttgart.de (C.L.); friedrich.gruener@mpa.uni-stuttgart.de (F.G.); juergen.frick@mpa.uni-stuttgart.de (J.F.); harald.garrecht@mpa.uni-stuttgart.de (H.G.)

**Keywords:** adobe, mud–straw plaster, ion chromatography, X-ray fluorescence, X-ray diffraction, sorption isotherm, thermogravimetric analysis, differential scanning calorimetry

## Abstract

In the restoration or rehabilitation of traditional buildings, compatible materials with known characteristics must be used. However, the existing literature lacks comprehensive studies on the characterisation of Persian mud–straw plaster, focusing primarily on Persian adobe. Moreover, previous research on Persian adobe has primarily employed XRF and XRD tests, neglecting ion chromatography, moisture sorption isotherm determination, and thermogravimetric analysis with differential scanning calorimetry. Consequently, there is a shortage of information regarding the elemental composition, mineralogical characteristics, moisture sorption behaviour, and thermal properties of Persian mud–straw plaster, as well as Persian adobe bricks. This paper aims to address this research gap by examining historical and new adobe bricks and mud–straw plaster used in Iran, utilising a comprehensive array of analytical techniques. The results from XRF analysis reveal relatively similar chemical compositions across all samples, while XRD analysis indicates predominantly similar mineral phases. Ion chromatography results demonstrate higher conductivity and chloride concentrations in the mud–straw samples than the adobe samples, with higher values for new samples than historical ones. Freshly used straw, clay, or soil may have higher chloride concentrations caused by the arid climate and soil salinisation in the area. Additionally, moisture sorption isotherm determination results show that adobe and mud–straw plaster with a higher salt load of chlorides have significantly higher moisture absorption. The increased straw quantity in the samples increases the moisture content. Furthermore, thermogravimetric analysis and differential scanning calorimetry indicate that, at low heating, adobe and mud–straw plaster lose water due to dehydration, and at high heating, they lose carbon dioxide due to decarboxylation. The comprehensive characterisation of Persian adobe and mud–straw plaster in this study fills a significant gap in the literature and offers invaluable insights for informing restoration and rehabilitation processes, ensuring the compatibility of the materials used.

## 1. Introduction

The construction materials used as the components and main elements of buildings have historically required extensive knowledge, study, and research. Advances in the construction of new materials necessitate a fundamental understanding of materials from the past. Adobe stands as one of the oldest construction materials in the world, renowned for its specific characteristics, such as distinguished hygrothermal properties, affordability, low energy consumption for production, environmental compatibility, and high resistance to heat and fire [1].

Traditional plaster, commonly used in adobe buildings, is mud–straw made from clayed earth and wheat straw mixed with water. Apart from its primary role in protecting the substrate, mud–straw plaster significantly influences the hygrothermal performance of the building, enhancing indoor air quality and comfort [2].

For traditional buildings, which often require continuous maintenance and periodic repair, restoration, or rehabilitation, any intervention plan involving materials must prioritise compatibility with the original materials. Different forms of compatibility, including chemical, physical, mechanical, rheological, and thermal compatibilities, must be carefully considered [3].

Statistics indicate the global prevalence of adobe and earth-based housing, emphasising the importance of preserving this longstanding tradition. In Iran alone, a significant portion of housing units are constructed using clay and mud materials, underlining the need for attention to earthen architecture on both historical and contemporary fronts [4].

Today, there is a growing trend towards strengthening and constructing earthen/adobe buildings, with the use of soil in architecture finding increasing applications not only in developing countries but also in developed regions such as the United States and various European countries [5].

In light of this, comprehensive knowledge of earthen materials and an understanding of their advantages and disadvantages are imperative [6].

Despite the historical significance and widespread use of adobe and mud–straw plaster, there remains a notable gap in the literature regarding the comprehensive characterisation of these materials that can be used in the restoration and rehabilitation of adobe buildings, particularly in the context of Persian architecture. Previous studies on Persian earthen materials have focused primarily on adobe bricks, with limited exploration of mud–straw plaster. Furthermore, the existing research has predominantly utilised XRF and XRD analyses, neglecting other critical aspects, such as ion chromatography, moisture sorption behaviour, and TGA-DSC analyses.

This paper aims to bridge this gap by conducting a thorough investigation into the characterisation of Persian adobe and mud–straw plaster for restoration and rehabilitation purposes. By exploring elemental composition, mineralogical characteristics, moisture sorption behaviour, and response to thermal analysis through techniques like TGA-DSC of both historical and new adobe bricks and mud–straw plaster used in Iran, this study contributes to a deeper understanding of these traditional building materials and their suitability for restoration and rehabilitation projects.

The knowledge gained from XRF and XRD analyses provides insights into the elemental and mineralogical composition of adobe and mud–straw plaster, which are crucial for understanding their structural integrity and durability over time. Additionally, ion chromatography can offer valuable information about the presence of soluble salts and ions within these materials, which can affect their long-term stability and compatibility with restoration interventions.

Understanding the moisture sorption behaviour of adobe and mud–straw plaster is essential for assessing their response to environmental conditions and potential risks of decay, such as efflorescence or salt crystallisation. This information can inform appropriate conservation strategies to mitigate moisture-related damage and ensure the long-term preservation of historical structures.

Furthermore, the knowledge gained from TGA-DSC analyses can significantly support restoration and rehabilitation interventions for adobe and mud–straw plaster in several ways. Firstly, TGA-DSC tests provide insights into the thermal decomposition behaviour of these materials, which is crucial for understanding how they respond to heat and fire exposure. This information can inform decisions related to fire safety measures and the selection of appropriate fire-retardant treatments during restoration efforts. Additionally, TGA-DSC analyses can help identify any organic and inorganic additives present in the materials, which may influence their structural integrity, durability, and susceptibility to degradation over time. Understanding the composition and thermal characteristics of adobe and mud–straw plaster through TGA-DSC testing allows for informed decision-making regarding material selection, conservation treatments, and preservation strategies, ultimately contributing to the long-term sustainability and resilience of historical adobe structures.

Therefore, the comprehensive characterisation of adobe and mud–straw plaster through techniques such as XRF, XRD, ion chromatography, and the assessment of moisture sorption and TGA-DSC analyses enhances the understanding of these traditional building materials and facilitates informed decision-making in restoration and rehabilitation projects aimed at preserving Persian architectural heritage.

**Figure 1:**
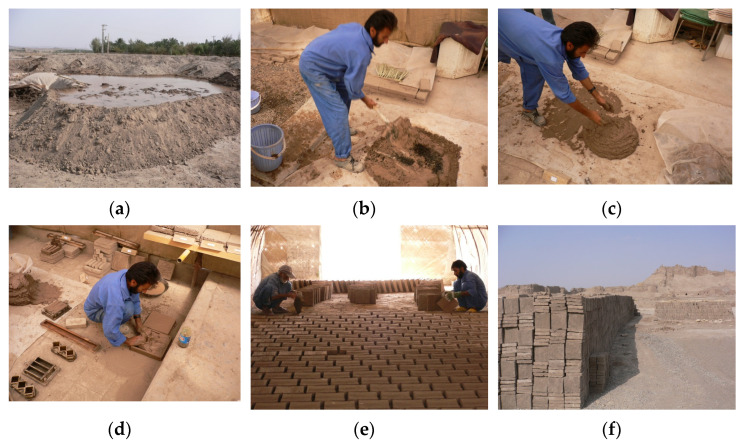
Adobe production: (**a**) adding water to soil and leaving for one or two days, (**b**) mixing adobe components using a shovel, (**c**) mixing adobe components by hand, (**d**) forging in the mould, (**e**) trimming and placing the adobe units vertically for drying faster, (**f**) dried adobe units ready to be used in construction, Bam citadel, 2007.

Figure 1 illustrates the traditional method of producing adobe bricks, hereafter referred to as adobe in this paper, in the Bam Citadel in Iran in 2007 during its restoration after the earthquake of 2003. To produce adobe, sand is mixed with clay. In some cases, fibrous materials are added to the mixture to prevent cracking during drying, increase tensile strength, enhance thermal insulation, or reduce weight. Fibrous materials include straw or the droppings of herbivores, such as goats, cows, horses, and camels. In Iran, adobe is generally made of clay soil containing 30% to 40% clay and 60% to 70% earth mixed with water, comprising approximately 25% of the weight of the dry soil. Typically, wheat straw is added to the mixture of clay and earth, constituting approximately 0.5% of the weight of dry soil [7] (Table 1). As shown in Figure 1, adobe can be made simply by forming the mixture and allowing it to dry in the sun. Water is added to clay and earth, left for one or two days to allow the earth mixture to soak in water to activate the clay, and mixed by a shovel or by feet, and then by hand. The mixture is then forged into moulds. After one or two days, the adobe units are placed vertically for faster drying. The dry adobe units are then collected together to be used.

**Figure 2:**
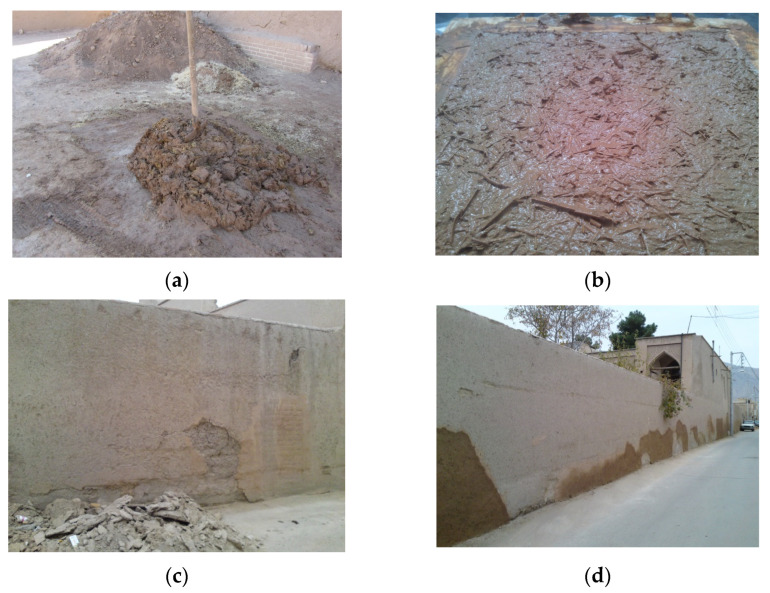
Mud–straw plaster production and usage: (**a**) mixing clay soil, straw, and water, (**b**) leaving mud–straw mix for one or two days, (**c**) removing the old mud–straw plaster, (**d**) plastering the adobe wall with new mud–straw plaster, Soukias adobe house (seventeenth century), Art University of Isfahan, Isfahan, 2013.

One of the most commonly used traditional plasters for covering adobe walls or roofs is mud–straw plaster, hereafter referred to as mud–straw in this paper. It is used as a thermal and moisture insulation layer. Adobe walls and roofs are protected by being plastered with a 2.5 cm to 3 cm thick layer of mud–straw. Mud–straw plaster is composed of clay, sand, water, and straw, typically wheat straw or grass. This plaster adheres to the adobe unit because both are made of the same materials. The straw content in mud–straw typically varies between 5% and 10% by weight. The water content for the mixture is about 25% of the weight of the clay soil (Table 1). Straw is cut into 5 cm lengths and added to the plaster. Figure 2 shows the phases of producing mud–straw plaster. Clay soil, straw, and water are mixed and left for two to four days. Then, the mud–straw is mixed again using a shovel or feet and placed on the surface of the adobe wall or roof to protect it.

This paper investigates the characterisation of Persian adobe bricks and mud–straw plaster for rehabilitating historical adobe buildings. To achieve this, specific historical adobe buildings with original materials from the same location were selected, and their samples were analysed. The samples consisted of two original historical adobe bricks, one new adobe brick used for restoration and rehabilitation, one sample of mud–straw plaster produced two decades ago, and another sample of mud–straw plaster produced in the laboratory using materials sourced from the same region.

After providing an introduction to the significance of these traditional building materials and their widespread use, the paper proceeds to a comprehensive literature review. Following this, the methodology section details the equipment used, sample preparation procedures, and analytical methods employed, including ion chromatography, XRD analysis, XRF analysis, determination of sorption isotherms, and thermogravimetric analysis with differential scanning calorimetry. The subsequent sections present the results obtained from these analyses, discussing the ion chromatography results, XRF and XRD tests, sorption isotherm results, and TGA/DSC results in detail. The discussion section further interprets these results, highlighting key findings and their implications. Finally, the paper concludes with a summary of the findings and their relevance to the rehabilitation of Persian adobe and mud–straw materials.

## 2. Literature Review

In selecting the previous research projects referenced in this section, careful consideration is given to their relevance to the research gap, scope, and objectives of this study. The first part of the literature review focuses on research worldwide concerning adobe and clay-based plasters, aiming to provide a comprehensive overview of studies relevant to traditional building materials. The second part specifically examines research conducted on Persian adobe, seeking to identify gaps in knowledge regarding this particular material. Each cited work is chosen based on its contribution to the understanding of elemental composition, mineralogical characteristics, moisture sorption behaviours, and thermal properties of adobe and clay-based plaster. By examining previous research endeavours, the aim is to establish a foundation for the study, identifying areas where gaps in knowledge persist and where the investigation can make a meaningful contribution.

Studying the characteristics of adobe and clay-based plasters has been the focus of a number of researchers worldwide. For adobe bricks, Coffman et al. [8] conducted a mineralogical study of adobe bricks from many historical adobe buildings in different parts of the world. They determined mineral composition, including clay type and quantity and soil granulation for adobe samples. They studied the effect of two chemical consolidants, an alkoxysilane and an isocyanate, on adobe samples and found that chemical consolidation depends very much on clay mineralogy and grain-size distribution.

Baglioni et al. [9] conducted experimental tests on rammed earth and adobe used in the Drâa Valley, Morocco, in order to determine the mineralogical and mechanical properties of the materials. They found that the earth used for adobe is richer in clay compared to that used for rammed earth. Both types of earth showed high cohesion and low expandability, making them quite suitable for construction. They contained quartz, feldspars, and a small amount of expandable clay minerals like smectite, which increase plasticity and cohesion but can also lead to shrinkage problems. Old adobe was more clayey than new adobe and, therefore, more suitable for construction.

Fratini et al. [10] studied the mineralogical, physical, and mechanical properties of adobe samples from seven different buildings in the historical centre of Lamezia Terme, Italy. They showed that adobe samples contained quartz, feldspars, clay minerals, and accessory minerals (micas and iron hydroxides). In most of the samples, the clay minerals were constituted by minerals, such as illite and kaolinite, that do not have swelling behaviour. Only in a few samples, clay minerals with swelling characteristics, such as chlorite-vermiculite and smectite, existed. Only one sample had a significant amount of calcite.

Costa et al. [11] studied the influence of the mineralogical composition on the properties of adobe bricks from various buildings in Aveiro, Portugal. Adobe samples were characterised by their mineralogical composition, soil granulation, water uptake, durability, and mechanical strength. Kaolinite, illite, and smectite were the main clay mineralogical compositions. They concluded that the phyllosilicate content, in particular kaolinite abundance, had a positive effect on the absorption/drying and mechanical properties of adobe bricks.

El Fgaier et al. [12] performed a hygroscopic analysis of sorption and desorption isotherms and studied the effect of sorption capacity on the thermo-mechanical properties of three types of unfired clay bricks industrially produced in northern France. The studied samples had higher sorption capacity than other construction materials, such as fire clay bricks. At 95% relative humidity, the moisture content of unfired clay bricks reached 3.5%. This phenomenon would make earthen materials able to balance the indoor climate by releasing or adsorbing moisture according to changes in the relative humidity of the ambient air. However, by increasing the relative humidity, the thermal capacity increased due to the penetration of water vapour and by acting as thermal bridges.

De Castrillo et al. [13] compared pre-historical and nineteenth- and twentieth-century traditional and contemporary adobe bricks in Cyprus. Tests were performed for physical and mineralogical characterisation of raw materials used in the production of adobe bricks. According to the chemical analysis and XRF test, the dominant chemical elements in adobe samples were calcium, silicon, iron, and aluminium. Calcium had the highest concentration, which could be related to the addition of lime during the mix design in order to improve the cohesion of the adobe brick. Silicon dioxide concentration was the second dominant element, attributed to the existence of quartz in the raw material for making adobe. The XRD test was consistent with the XRF results, indicating calcite, quartz, and albite as the dominant minerals in all adobe samples. Some adobe samples had high amounts of gypsum attributed to the gypsiferous soils of the region. In general, irrespective of the period, calcite, quartz, and albite were the dominant minerals in all adobe samples. Some certain differences in the chemical composition of the adobe samples, such as high amounts of gypsum in some samples, were attributed to the type of soil of the region.

Ashour et al. [14] determined the equilibrium moisture content (EMC) of mud–straw plaster. The earth used had four different compositions of cohesive soil and sand. Three types of fibre materials, wheat straw, barley straw, and wood shavings, were added to the soil. The results show that the EMC increases with an increase in relative humidity and decreases with an increase in temperature. Additionally, the effect of relative humidity on the EMC is more pronounced than that of temperature. For a relative humidity of 43%, the EMC for different samples was approximately 1.1% to 3.6% at 10 °C and about 0.8% to 2.9% at 40 °C. By increasing the humidity to 95%, the EMC increased to approximately 2.3% to 5.5% at 10 °C and 1.9% to 4.8% at 40 °C.

Costa et al. [15] characterised adobe samples taken from the central coastal region of Portugal. Adobe samples were divided into four groups according to their mineralogical composition: lime-stabilised adobe with a high percentage of calcite, solid adobe with a high amount of phyllosilicates, medium adobe with similar quartz and calcite contents, and iron-rich adobe with higher values of iron oxides and hydroxides than the others. In general, adobe samples consisted mainly of quartz, calcite, and phyllosilicates. Quartz, calcite, phyllosilicates, and K-felspars were the main minerals of the silt–clay fraction of the adobe samples. Iron oxides and hydroxides dominated the accessory minerals. The phyllosilicates identified were clay minerals like kaolinite, illite, smectite, chlorite, vermiculite, and illite–smectite. The mineralogical composition of the adobe samples was mainly composed of silica, calcium, and aluminium.

Sanchez-Calvillo et al. [16] characterised adobe samples from damaged buildings in Jojutla de Juarez, Mexico. Mineralogical and granulometry analyses were performed. Calcite was present in most of the adobe samples. Kaolinite with high proportions was observed in a number of adobe samples. The cause of the use of kaolinite in producing adobe bricks was attributed to its low swelling and shrinkage properties.

Laborel-Préneron et al. [17] studied the hygrothermal characteristics of earthen materials containing a large volume, about 40% of the volume corresponding to about 6% of weight, of the plant aggregates of barley straw, hemp shiv, or corn cob. The results show a low thermal conductivity with this large volume of plant aggregates. The water vapour permeability did not improve. The sorption–desorption isotherms indicated that the sorption capacity improved only slightly due to the low plant matter mass.

Gomes [18] investigated the hygrothermal characteristics of earthen materials. The sorption and desorption curves were obtained at three oven-drying temperatures. The maximum water content ranged from 3% to 5%.

Mellaikhafi et al. [19] performed physical–chemical, mineralogical, and geotechnical tests on five types of soils used for making rammed earth and adobe in an oasis of south-eastern Morocco. The main minerals in the soils were quartz, calcite, ferroan, clinochlore, and muscovite. Two clay minerals, kaolinite and illite, were absent. Two minerals, smectite and vermiculite, with swelling properties, were also absent.

For earth-based plaster, Ranesi et al. [20] studied the relative humidity-related properties of five plastering mortars with clay, air lime, and natural hydraulic lime bases and three finishing pastes with gypsum and gypsum–air lime bases, in addition to cement-based plaster for comparison. The earth plaster was the most appropriate plaster for relative humidity passive regulation and showed high hygroscopicity. Thereafter, the combination of gypsum and air lime was more suitable than the pure gypsum paste, which showed a very low moisture capacity. The adsorption/desorption of the natural hydraulic lime mortar was moderately good. Hydrated air lime plaster had the lowest adsorption and desorption. The cement plaster was the least suitable, with the lowest water vapour permeability and slow adsorption and desorption.

Lima et al. [21] examined the effect of clay mineralogy on earth plaster properties. Three mortars were produced using different clayish earths to assess their influence. The results show that clay mineralogy significantly impacted plaster properties, such as vapour adsorption, drying shrinkage, mechanical strength, dry abrasion, and thermal conductivity. Illitic clayish earth exhibited balanced properties, making it suitable for earth-based plasters.

Savadogo et al. [22] investigated the physico-mechanical and durability properties of earthen plaster stabilised with fermented rice husk. Various mixtures were tested, revealing improvements in properties with the addition of fermented rice husk. However, excessive rice-husk content negatively affected water absorption and erosion resistance, suggesting an optimal ratio for enhanced performance.

Santos et al. [23] evaluated the efficiency of earth plaster on different masonry types. A commercial unstabilised earth mortar was used to plaster experimental masonry walls, demonstrating durability across various substrates. Despite initial variations, earth plaster exhibited long-term stability, proving its technical efficiency across historical and contemporary masonry structures.

Vares et al. [24] assessed the hygrothermal performance of clay–sand plaster. Different covering materials were tested to evaluate the moisture buffering and vapour-permeability properties. The results indicate variations in moisture uptake and diffusion, emphasising the importance of material selection for indoor climate control.

Santos and Faria [25] characterised various earthen plasters using laboratory and in-situ tests. Different mortar formulations were evaluated for mechanical properties and durability. The results highlight the influence of additives on plaster performance, with some formulations exhibiting improved adhesive strength and mechanical properties.

Santos et al. [26] conducted a comparative analysis of the mineralogical, mechanical, and hygroscopic properties of earthen, gypsum, and cement-based plasters. Five different plastering mortars were examined, including unstabilised and stabilised earth-based plasters, as well as gypsum and cement-based pre-mixed plasters. While earthen mortars exhibited lower mechanical strength compared to gypsum and cement-based mortars, they demonstrated the highest hygroscopicity, functioning as passive moisture buffers.

Ojo et al. [27] explored the characteristics of unfired earthen building materials using muscovite-rich soils and alkali activators. They discovered that alkali activation significantly improved the physical and mechanical properties of these materials, suggesting a potential for sustainable low-cost housing solutions.

Bass et al. [28] examined ancient earthen plaster from three Native American sites in the American Southwest. Their analysis studied the microstructure, mineral composition, and deterioration mechanisms. Their research reveals how locally sourced plaster was tailored to suit specific site conditions and functional requirements, highlighting the adaptability of ancient plaster technologies.

Muşkara and Bozbaş [29] characterised earthen building materials in vernacular houses in North-West Turkey, focusing on the Saraylı, Örcün, and Selimiye villages. They conducted archaeometric investigations on samples from earthen bricks, mud plasters, and mortars, analysing their mineralogical and chemical properties. The study aimed to understand building technologies and raw material properties for developing restoration strategies aligned with sustainable architecture principles.

Saleh [30] investigated natural adobe plaster in the Jordan Valley, using advanced techniques to analyse plaster morphology. The results reveal significant variance in plaster recipes across different regions, suggesting local soil differences or additive variations. Additionally, it was found that regardless of local recipes, three layers were typically used for exterior wall protection, each with different ratios of earth materials.

Saleh [31] conducted an experimental campaign to characterise wall plaster in Al-Ulla, Saudi Arabia. Various methods, including visual inspections, microscopic analysis, advanced imaging techniques, and mineralogical analysis, were employed. Four types of plaster were identified through the experiments, which facilitatedthe accurate diagnosis of conservation strategies for Qarh’s monuments at Al-Ulla.

Silveira et al. [32] conducted studies on rehabilitating a cultural heritage district with traditional adobe constructions in the Aveiro district, Portugal. Their research efforts focused on characterising adobe’s composition, mechanical behaviour, and structural performance to guide rehabilitation practices effectively. Based on their findings, they suggested specific methods for improving adobe composition to enhance durability and seismic resistance. Additionally, they provided recommendations for structural reinforcement techniques tailored to the unique characteristics of traditional adobe constructions.

Sánchez et al. [33] investigated various physical and mechanical properties of adobe for the rehabilitation of adobe buildings. They conducted a comprehensive review of experimental studies spanning the last 15 years from different countries to examine the mechanical properties of adobe masonry. By comparing the results of these studies, correlations between different physical and mechanical properties were established and compiled in their document, providing valuable insights for adobe building rehabilitation efforts. However, their review revealed significant variations in regulations to accommodate local conditions, highlighting the need for adaptable guidelines in the field.

Papayianni and Pachta [34] studied the consolidation and upgrading of historical earth block masonry constructions. They presented a methodology for analysing the building materials and techniques of historical earth block houses in northern Greece, alongside the design and testing of compatible repair materials for their rehabilitation. Laboratory tests were conducted on historical materials to determine their microstructural, physico-mechanical, and chemical properties. The results informed the development of compatible repair materials based on earth, including soil-based grouts with enhanced properties through specific additives and admixtures.

González-Sánchez et al. [35] investigated stabilised earthen mixtures to preserve traditional adobe buildings, focusing on enhancing durability for rehabilitation. Their study evaluated an earthen mixture with a vegetal origin gel from rice starch, aiming to improve mechanical strength and waterproofing. Divided into experimental and application phases, the research first developed eco-friendly mixtures and then applied them in adobe dwellings in Santa Ana Chapitiro, Michoacan, Mexico, through participatory design. Initial observations suggest promising early stage performance.

Faria et al. [36] conducted an experimental characterisation of an earth plastering mortar for the rehabilitation of adobe buildings. An extended experimental campaign was developed to assess multiple properties of a ready-mixed earth plastering mortar and to increase scientific knowledge of the influence of test procedures on those properties. The plaster satisfied the requirements of the existing German standard and seemed adequate for application as rehabilitation plaster on historical adobe buildings.

Gomes et al. [37] investigated the compatibility of earth-based repair mortars with rammed earth substrates. The study analysed the performance of eight repair mortars formulated with earth collected from rammed earth buildings in South Portugal or a commercial type of earth and tested with four types of binders. Mortars were applied on two standard defects on rammed earth blocks, representing common issues on exterior surfaces. The study evaluated mortar performance, substrate compatibility, and visual effectiveness of the intervention. The results show variations in mortar behaviour based on the type of rammed earth support, with unstabilised earth mortars demonstrating the best performance compared to their stabilised counterparts.

Jia et al. [38] analysed historical earthen plaster to enhance its characteristics for restoration. They investigated the effects of different material compositions on shrinkage and cracking properties through laboratory tests on plaster specimens. The results show that adding sand and vegetal fibres improved plaster properties by increasing the shrinkage limit and inhibiting volume shrinkage. However, wheat straw led to surface cracking, while calcined ginger nut paste displayed potential for plaster restoration with minimal shrinkage and surface cracking. The study aimed to understand the scientific nature of earthen plaster and its behaviour during desiccation.

Mattone et al. [39] conducted experimental tests on earth–gypsum plasters for the conservation and rehabilitation of earthen constructions. The study aimed to develop effective materials to protect earthen architectural heritage from weathering, considering the significance of earthen architecture worldwide. By mixing earth and gypsum with natural or synthetic additives, the research sought to design plasters capable of withstanding atmospheric agents prevalent in various locations where these constructions are situated.

A few investigations have been performed on the characteristics of Persian adobe. Hosseini et al. [40] conducted mineralogical and physical tests on two historical and two new adobe samples from Belqeis Castle in Esfarayen, North-East Iran. The results show that a part of the minerals included calcite and quartz, indicating a lack of clay minerals. In addition, the majority of minerals were silicates (muscovite, biotite, feldspar, and enstatite).

Zakavi [41] studied the soil from six mines near the Choga Zambil in Susa, South-East Iran, for making new adobe bricks. Calcite and quartz were the dominant minerals in the soil samples. The soil samples were poor in terms of high-quality clay minerals such as kaolinite and montmorillonite. The amount of chlorine and sulphate ions in four soil samples was high. Soil samples containing sodium and potassium chloride showed a higher potential for swelling of the clay.

Dormohamadi and Rahimnia [42] studied dynamic compaction on the mechanical behaviour of adobe bricks made with six different clayey–silty soil types from six different mines in the town of Ardakan, near Yazd, Central Iran. The experiments included the determination of physical, mineralogical, and chemical characterisation, as well as mechanical properties tests. The results show that the main component of all six soil samples was a mixture of silicates and calcium aluminates (feldspar) resulting from the combination of silica with lime, alumina plus magnesium oxide, and iron oxide. In all of them, the amount of silica was approximately one-third of the weight of the soil. This is due to the high amount of aeolian sand in this desert region of Iran. The amount of alumina was less than the amount of silica in all samples. The four main phases of mineralogical composition, i.e., quartz, calcite, feldspars, and clay minerals, as well as traces of dolomite were present in all soil types. The soils were rich in silt and silica due to the high amount of aeolian sand, again a characteristic of the soil of the region. The amount of swelling clay minerals, like smectite, in the soil was low.

Eskandari [43] studied the physical, mineralogical, and mechanical properties of Persian historical and new adobe bricks. Six groups of adobe bricks, consisting of three groups of historical adobe bricks from the town of Maybod, near Yazd, and from the town of Jarquyeh, near Isfahan, and three groups of new adobe bricks from Maybod and Yazd, Central Iran, were tested. The results show that quartz was a significant amount of the constituent minerals. These results are also consistent with the geological characteristics of the soil formation in the desert areas of the studied adobe bricks and the presence of abundant wind sand in those areas. Calcite was the second most abundant mineral in the soil of each study group, which is due to the characteristics of arid and semi-arid regions. Albite was the third most abundant mineral among the studied adobe bricks groups, which provides relatively good resistance to adobe bricks.

Table 2 summarises the important findings based on characterising the adobe and clay-based plaster samples.

The review of the literature indicates that there has been no study on the characterisation of Persian mud–straw, and the studies focused only on Persian adobe. Even in the case of Persian adobe, only XRF and XRD tests have been conducted. There has been no ion chromatography, moisture sorption isotherm determination, thermogravimetric analysis, or differential scanning calorimetry reported for Persian adobe bricks. Therefore, in the existing literature, there is no information about the elemental composition, mineralogical characteristics, moisture sorption behaviour, and thermal properties of Persian mud–straw plaster. In addition, the nonexistence of information about the moisture sorption behaviour and thermal properties of Persian adobe bricks provides an important gap in knowledge about Persian adobe. This paper examines the above-mentioned characteristics of both historical and new adobe bricks and mud–straw plaster produced and used in Iran in order to bridge the existing gap.

## 3. Samples, Method, and Equipment

### 3.1. Samples

**Figure 3:**
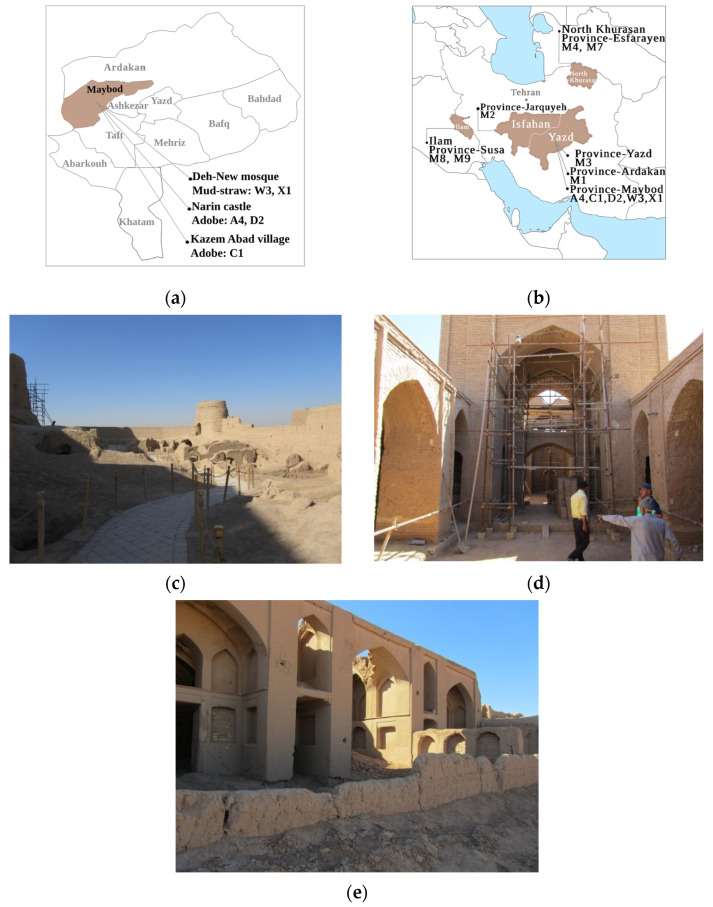
Locations of studied adobe and mud–straw samples: (**a**) in this study (Narin Castle and Kazem Abad village in Maybod) with sample codes, (**b**) by other researchers (Yazd, Ardakan, Esfarayen, Jarquyeh, and Susa) and in this study (Maybod) with sample codes, (**c**) Narin Castle, (**d**) Deh-Naw Mosque, (**e**) Kazem Abad village.

Adobe bricks and mud–straw samples for testing were taken from different adobe buildings in the town of Maybod, 55 km west of the city of Yazd, Central Iran (Figure 3). The locations and periods of the samples, along with their respective straw content and corresponding geology region, are provided in Table 3. The selection of locations was based on the availability of historical buildings constructed with original adobe and mud–straw in the Maybod region. These locations were chosen to encompass a range of construction practices and historical periods representative of Persian architecture. The samples were carefully chosen to ensure the representation of both recent restorations and original structures, thereby providing a comprehensive overview of the materials used in historical building construction in the area. The number of samples was limited to ensure a manageable scope for the study while still capturing the diversity of materials and construction practices found in Persian architecture. Additionally, the dimensions of each sample are included in the table to provide further context regarding their physical characteristics and construction methods.

**Figure 4:**
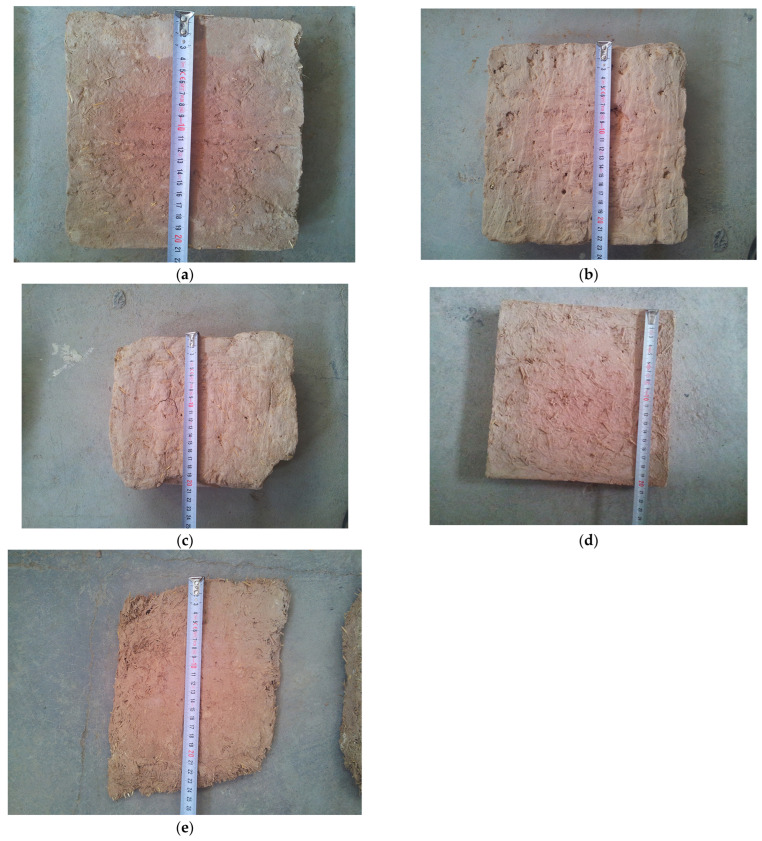
Tested adobe and mud–straw plaster samples: (**a**) adobe brick A4 (Narin Castle, Maybod, 2017), (**b**) adobe brick C1 (Kazem Abad village, Maybod, 1870), (**c**) adobe brick D2 (Narin Castle, Maybod, 1320), (**d**) mud–straw plaster W3 (Deh-Naw Mosque, Maybod, 2019) made in the laboratory, (**e**) mud–straw plaster X1 (Deh-Naw Mosque, Maybod, 2009).

The town of Maybod has an arid and desert climate. The adobe bricks tested are coded as follows: A4, with dimensions of 21 × 21 × 5.4 cm^3^, made in 2017 in Narin Castle and used for recent restorations; C1, with dimensions of 22.5 × 23 × 6 cm^3^, made in 1870 in the Kazem Abad village as an original and historical adobe brick; and D2, with dimensions of 20 × 24.5 × 5.5 cm^3^, made in 1320 in Narin Castle as an original and historical adobe brick. The mud–straw plasters tested are coded as follows: W3, with dimensions of 20 × 20.5 × 2.7 cm^3^, made in 2019 in the laboratory for the present study from materials used for the restoration of the Deh-Naw Mosque, and X1, with approximate dimensions of 24 × 18 × 1.5 cm^3^, made in 2009 in the Deh-Naw Mosque used for previous restorations (Figure 4, Table 3). Due to limited access and the availability of samples, particularly historical ones, only one sample of each type of adobe brick and mud–straw plaster was tested. The measurement of straw content in the adobe and mud–straw samples revealed that the adobe samples A4, C1, and D2 contained 0.58 wt%, 0 wt%, and 0.02 wt% straw, respectively, while the mud–straw samples W3 and X1 contained 11 wt% and 8 wt% straw, respectively. Other researchers, as described in Section 1, have studied a number of adobe samples from other parts of Iran, including Yazd, Maybod, Ardakan, Esfarayen, Jarquyeh, and Susa (Figure 3a), whose results will be compared with results of this study in following sections.

### 3.2. Geology of the Region

**Figure 5:**
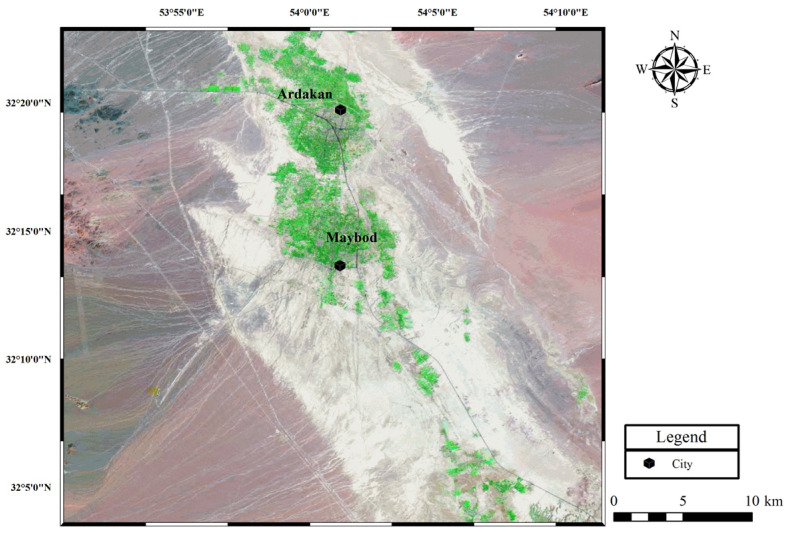
Satellite map of the region [45].

Since the construction materials used in traditional buildings are often supplied from the nearest suitable location, the soil and rock materials around the site should be carefully studied. According to Ghorbani [44], the region from which the materials are taken is located in the structural zone of Central Iran. The area around the towns of Maybod and Ardakan is surrounded by the heights of the region. The heights on the eastern side are composed of Eocene formations. Figure 5 shows the satellite map of the region created from a Landsat ETM+ [45] satellite image created by the authors.

**Figure 6:**
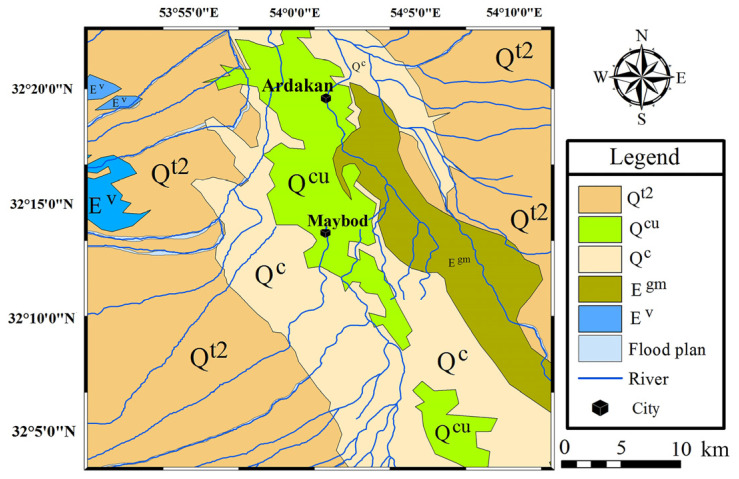
Geological map of the region.

The heights on the west side are composed of tuff, igneous units, such as Eocene andesitic and dacitic lavas and trachyte and rhyolite lavas, as well as Palaeozoic era (Cambrian period) and black limestone. Erosion by wind and seasonal floods has caused erosion of the weaker Neogene and Eocene units, and as a result, the sediments created in the plains of the region have been deposited and caused land terraces. The towns of Maybod and Ardakan are located on the sediments of the present era, which include clay flat (Q^c^ unit) and cultivated lands (Q^cu^ unit). Figure 6 shows the geological map of the region prepared from satellite data and combining the existing geological maps [46,47].

It should be noted that adobe bricks are made of fine-grained soil, but different soil types have various properties that affect the quality of adobe bricks. So, the origin and geological process for the deposition and composition of soil are important. In the following, the general geological characteristics of the geological units of the region shown in Figure 6, created by the authors, are described.

#### 3.2.1. Gypsiferous Marl (E^gm^)

This unit from the Eocene epoch is located on the unit of sandstone, marl, shale, tuff, and gypsum or unit E^s,m^. The material of this unit is clay shale (mudstone), which is brick-coloured, with layers ranging from red to brown clay, and thin gypsum interlayers can be observed between them [48]. In this unit, conglomerate, red-to-brown sandstone layers can be seen, and the protrusions of these layers show the trend of the layers in this region. The main volume of this unit in the range of the map in Figure 6 is made of shale, and conglomerate layers are seen as interlayers in it, which indicates the successive changes in energy and depth of the basin at the time of the sedimentation of this unit. For most of the deposition time of this unit, a calm environment and moderate depth have been dominant. The red colour of this unit indicates the oxidation conditions in the formation environment of this unit. Also, hot and dry weather conditions must have been established in the region. In addition, the depth of water and the energy of the environment had been such that it has made it possible for oxygen to be in contact with the sediments that were forming. The exact age of this unit is attributed to the Middle Eocene (Lutetian) according to the identified fossils.

#### 3.2.2. Lavas and Tuffs (E^v^)

This unit mainly consists of grey and, sometimes, green andesitic, dacite, and rhyodacite tuffs and lavas. The texture of these rocks in the lava section is porphyry, and in the tuff sections, it is clastic and vitroclastic. The plagioclase minerals of these rocks are decomposed into epidote, chlorite, sericite, and clay minerals, and its amphiboles are decomposed into epidote, tremolite, and chlorite. Quartz mineral phenocrysts are also found in these rocks. The chemical composition of two rock samples of this unit contains the oxides presented in Table 4 [49].

#### 3.2.3. Young Terraces and Gravel Fans (Q^t2^)

This unit is a new alluvial sediment that forms most of the loose flat plains. It consists of rock fragments that are older than the Quaternary formation and eroded fragments of the Q^t1^ unit (Pliocene epoch). Its matrix consists of clay, silt, and sand and is free of cement. This unit is composed of a larger grain size near the heights and is added to the middle of the plains by moving away from the heights of the larger parts and towards its clay, silt, and sand. The slope of the layering of this unit is horizontal. The age of this unit is Holocene or the present day.

#### 3.2.4. Clay Flat (Q^c^)

This clay soil unit is equivalent to the Q^t2^ unit that forms the young garrisons and alluvial fans. Due to the distance of Q^c^ from the heights, its coarse-grained particles are reduced, and its main volume is fine-grained, such as clay and silt particles. This unit forms flat and smooth lands, and agricultural lands are often close to this unit.

#### 3.2.5. Cultivated Land (Q^cu^)

This agricultural land unit is actually part of the Q^t2^ unit that can be cultivated. Therefore, areas of the Q^t2^ unit that, due to the distance from the heights, their coarse grains are reduced, and the volume of sand, silt, and especially clay particles are increased are the so-called Q^cu^. This unit is found adjacent to the Q^c^ unit in the region.

### 3.3. Method

Before providing a detailed explanation of each method employed in this study, it is essential to provide a general overview of the rationale behind the selection of these methods and their significance in addressing the research objectives. The characterisation of adobe and mud–straw samples necessitates a comprehensive approach that encompasses various analytical techniques to gain insights into their elemental composition, mineralogical characteristics, moisture sorption behaviours, and thermal properties. The chosen methods offer distinct advantages in explaining the structural, compositional, and thermal attributes of these traditional building materials. Additionally, reference will be made to previous studies that have analysed similar samples to provide context for the comparisons made throughout the paper.

#### 3.3.1. Rationale for Method Selection and Standards

The methods employed in this study were carefully chosen based on their ability to provide detailed insights into different aspects of adobe and mud–straw samples. Ion chromatography, performed according to DIN EN ISO 10304-1 [50] and DIN EN ISO 10304-4 [51], facilitates the quantification of salts present in the samples, which is crucial for understanding their deterioration mechanisms and informing conservation strategies. X-ray diffraction (XRD) analysis, according to DIN EN 13925-2 [52], offers valuable information about the mineralogical composition and crystalline phases present in the samples, aiding in the identification of key mineral constituents. X-ray fluorescence (XRF) analysis, according to DIN 51001 [53], complements XRD by providing data on the elemental composition of the samples, enhancing our understanding of their chemical characteristics. The determination of hygroscopic sorption properties through gravimetric analysis, according to DIN EN ISO 12571 [54], offers insights into the moisture sorption behaviour of the materials, which is essential for assessing their durability and performance in different environmental conditions. Thermogravimetric analysis (TGA), according to ISO 11358-1 [55], and differential scanning calorimetry (DSC), according to ISO 11358-1 [56], enable the investigation of thermal decomposition processes and phase transitions in the samples, describing their thermal stability and behaviour at elevated temperatures.

The selection of characterisation methods in this study was guided by a thorough review of the available literature, which offers a comprehensive overview of different techniques and their applications across various materials. The references [57,58] provide insights into the advantages and shortcomings of different characterisation techniques, aiding in the rationale behind their selection for specific analyses. While a multitude of methods exist for material characterisation, the chosen techniques were considered suitable for their ability to provide relevant information about the adobe bricks and mud–straw plasters under investigation.

#### 3.3.2. Sequence of Steps in Methods

Each method employed in this research follows a sequence of steps according to the specific analytical technique utilised. Ion chromatography, a method crucial for evaluating the presence and concentration of damaging salts in historical building materials, involves several sequential steps. Initially, samples containing straw are dried, crushed, and homogenised. Distilled water is then added for the elution process, followed by filtration. The soluble salts are determined by analysing the amounts of soluble ions in the solution. X-ray diffraction (XRD) involves multiple sequential steps for accurate mineralogical analysis. Initially, straw is removed from the samples to prevent interference during high-temperature analysis. The samples are then ground to approximately 5 µm, prepared on specific sample holders, and placed in the spectrometer. X-ray fluorescence (XRF) analysis requires meticulous sample preparation to ensure accurate chemical composition analysis. Sample preparation involves grinding the sample to a fine powder, mixing it with a binding/grinding aid, and pressing the mixture into a homogeneous sample pellet or using a suitable flux and heating it to make a fused tablet. The determination of hygroscopic sorption properties involves specific steps to obtain isotherm sorption curves. Samples are dried to a constant mass and then placed in the test device at a constant temperature, with relative humidity increasing or decreasing at steps. Moisture content is determined at each relative humidity, and the isotherm sorption curve is drawn accordingly. Thermogravimetric analysis (TGA) and differential scanning calorimetry (DSC) require precise sample preparation and testing procedures. Before conducting TGA and DSC, straw is removed from the samples to prevent potential interference or combustion effects at elevated temperatures. Samples are then prepared in two groups: completely dry and saturated. The samples undergo controlled temperature changes, and mass loss or heat flow is measured and recorded.

#### 3.3.3. Equipment

The devices for ion chromatography were Metrohm (881 Compact IC Pro for cations with 863 Compact Autosampler, Herisau, Switzerland) and Dionex (Dionex ICS-1500 for anions with Dionex AS-DV autosampler, Sunnyvale, CA, USA). The chemical composition of the soil samples was determined by XRF analysis, using a Bruker AXS S4 Pioneer spectrometer with Rh-radiation (Bruker AXS GmbH, Karlsruhe, Germany). Melting tablets were prepared, and the loss of ignition (1000 °C) was determined. Major elements and some trace elements were analysed. The mineralogical composition of the adobe samples was determined by X-ray diffraction using a Bruker AXS D8 Advance X-ray diffractometer (Bruker AXS GmbH, Karlsruhe, Germany) with a Cu anode, operating at 40 kV—30 mA, a step size = 0.01°, and a scanning range 2θ between 5° and 70° (Bruker AXS GmbH, Karlsruhe, Germany). The equipment used for the determination of the hygroscopic sorption properties was POROTEC GraviSorp 120 (POROTEC GmbH, Hofheim am Taunus, Germany).

### 3.4. Ion Chromatography

Knowledge of the quantitative concentrations and enrichment of damaging salts in all types of inorganic historical building materials is very important. Ion chromatography is a highly sensitive and fast method to evaluate a set of interesting and common salts in historical building materials. It allows for the separation of ions and polar molecules based on their interactions with the resin (stationary phase) and the eluent (mobile phase). The system employs a stationary phase, such as an ionic resin, packed in the column, and a mobile phase, typically an eluent made of sodium hydrogen carbonate with additional neutralising strippers for chemical suppression to remove all background eluent ions. This process decreases the conductivity of the eluent, facilitating the injection of the sample into the mobile phase for analysis. In ion chromatography, conductivity values are derived from the quantitative concentrations of ions present in the samples. Through the analysis of ion concentrations, conductivity levels can be determined using established methods or standards, allowing for comparisons between different samples. This method provides valuable insights into the conductivity characteristics of historical building materials, aiding in the assessment of their properties and potential salt enrichment. Ion chromatography is a powerful tool for separating and determining low concentrations of ions and is particularly useful in environmental and water quality studies [59].

The samples containing straw for ion chromatographic analysis are first dried to a constant weight and then crushed and homogenised. Distilled water is added for the elution process and later filtered. The soluble salts are determined by analysing the amounts of soluble ions in the solution. Different columns and eluents are used for cations (Na^+^, NH_4_^+^, K^+^, Mg^2+^, and Ca^2+^) and anions (F^−^, Cl^−^, Br^−^, NO_3_^−^, NO_2_^−^, PO_4_^2−^, SO_4_^2−^, and C_2_O_4_^2−^). Certified reference solutions with specific amounts of interesting ions were used for calibration in this study, and the dried samples were weighed first. Then, distilled water was added in a ratio of approximately 1:10, and the samples were weighed again. An ultrasonic bath was provided for 15 min. The samples were allowed to settle for more than 12 h. Then, the solutions were filtered and analysed.

### 3.5. XRD Analysis

XRD is a vital technique for characterising materials, utilising X-ray wavelengths ranging from 0.01 nm to 10 nm. It works in conjunction with XRF, which provides elemental data. XRD identifies and quantifies minerals and their species, revealing crystalline phases and offering comprehensive insight into chemical composition and crystal structure. Each crystalline structure generates a unique X-ray pattern, similar to a fingerprint for identification. XRD is adept at distinguishing between compounds, such as different oxidation states or polymorphs [59]. Sample preparation for X-ray powder diffraction involves removing straw to prevent interference during high temperatures, grinding the samples to approximately 5 µm, and placing them in specific sample holders. This technique provides valuable information on crystallographic structure and chemical composition, aiding in the identification of crystalline phases, even in compounds, and potentially harmful mineralogical phases, such as salts.

### 3.6. XRF Analysis

XRF offers insights into the chemical composition of samples, focusing on elemental analysis rather than specific phases. Utilising an XRF spectrometer, fluorescent radiation emitted by various atoms in the sample is measured to identify and quantify material elements. This technique provides detailed information on the elemental composition, including the presence and quantity of elements like Fe and O, presented as percentages or parts per million (ppm) in a graphical output [59]. Sample preparation involves grinding the sample to a fine powder, ideally less than 75 µm, and forming a homogeneous sample pellet through pressing or fusion with flux at high temperatures.

In this study, straw was first removed from the sample to ensure accurate XRD mineralogical analysis, preventing potential organic material interference. Then, the sample was ground and dried in a drying chamber at 105 °C. The dried sample was weighted precisely (at least 1.5 g) in a constantly heated porcelain crucible and calcined at 1000 °C in a muffle furnace. Next, 1.2 g of the sample, which had been calcined at 1000 °C, was weighed into a platinum crucible. For this purpose, 6 g of Spectromelt A12 (flux, Merck, Darmstadt, Germany) was weighed out accurately. Both substances were mixed with a spatula. The mixture was melted at 1150 °C for 20 min. The melt was swirled about every 5 min in order to remove air bubbles and for homogenisation. The melt that was as free of air bubbles as possible was transferred into a platinum mould. The mould was heated for at least 5 min before the transfer. The melt tablets were analysed with a Bruker AXS S4 Pioneer spectrometer with Rh-radiation (Bruker AXS GmbH, Karlsruhe, Germany) regarding their chemical element composition, i.e., their major and trace elements.

### 3.7. Determination of Sorption Isotherm (GraviSorp)

Hygroscopicity refers to a material’s ability to absorb moisture from its surroundings, while sorption capacity indicates its capability to absorb or release water vapour until reaching equilibrium. The relationship between moisture content and relative humidity at a constant temperature is represented by the moisture sorption isotherm or the isotherm sorption curve [60].

To obtain the isotherm sorption curve, the sample is dried to a constant mass and then placed in the test device at a constant temperature, with relative humidity increasing in steps. The moisture content is determined at each step after the sample achieves equilibrium, allowing for the construction of the isotherm sorption curve.

Similarly, to draw the isotherm desorption curve, the same procedure is followed but with a decrease in relative humidity. The starting point is a relative humidity of at least 95% or, alternatively, the last point of the sorption curve. At a constant temperature, the sample is placed in the test device, with the relative humidity decreasing in steps. By determining the moisture content at each relative humidity, the isotherm desorption curve can be drawn.

In the GraviSorp 120 from Porotec used for this research, 10 samples can be examined simultaneously under constant temperature and with predefined humidity levels

In this study, two sets of tests were conducted. The first set involved examining the sorption isotherm characteristics of adobe and mud–straw samples over a 30-day period. The second set of tests was exclusively conducted on mud–straw samples, both with and without the addition of straw, for a duration of 60 days. The second set aimed to investigate the influence of straw on the sorption isotherm performance of mud–straw samples.

All tests were conducted at a constant temperature of 22 °C, following a similar procedure. For instance, in the 30-day test, the relative humidity was incrementally increased from 10% to 95% in steps of 10% each, with each humidity level applied for 24 h during the first 15 days. Subsequently, during the latter 15 days, the relative humidity was gradually decreased from 95% to 10%. At each specific relative humidity level, the moisture content of the sample was measured. The point at which the sample reaches equilibrium with its environment, exchanging an equal amount of absorbed and desorbed water molecules, defines the moisture content known as the equilibrium moisture content (EMC). It is calculated from Equation (1), in which *W_m_* and *W_d_* are the moist and dry weights of the sample, respectively.

(1) $$EMC=\frac{(W_{m}-W_{d})\times 100}{W_{d}}$$

### 3.8. Thermogravimetric Analysis (TGA) and Differential Scanning Calorimetry (DSC)

Thermogravimetric analysis (TGA) is a thermal analysis method used to measure the mass changes of a material with respect to temperature changes. In the TGA test, the sample is heated, and its mass changes are continuously recorded to measure the mass loss relative to the initial mass of the samples. Thermogravimetric analysis provides information about the physical characteristics of materials, such as thermal decomposition and solid–gas reactions, including oxidation, reduction, and dehydration. Differential scanning calorimetry (DSC) is another thermal technique used to investigate characteristics such as heat capacity and chemical reactions, like the oxidation behaviour of materials. This is achieved by measuring the heat flow into or out of a sample as it undergoes physical and chemical changes during controlled temperature changes [61].

In this study, prior to conducting the TGA and DSC analyses, straw was removed from the samples to prevent potential interference or combustion effects at elevated temperatures. Subsequently, two groups of samples were prepared from each of the three types of adobe and two types of mud–straw samples: one group of completely dry samples and another group of saturated samples. The dry samples were exposed to silica gel in a sealed container for four months until they reached complete dryness, as indicated by no further change in weight during periodic weighing. Conversely, the saturated samples were placed in a humidity chamber for four months until they reached saturation, confirmed by stable weights during periodic weighing. Following the preparation of these samples, an STA 409 PC Luxx Simultaneous thermal analyser (Netzsch-Gerätebau GmbH, Selb, Germany) was utilised for simultaneous TG-DSC measurements. During the test, the temperature was incrementally increased from 25 °C to 980 °C according to a controlled temperature program, and the mass loss of the samples was measured and recorded.

## 4. Results and Discussion

This investigation focused on traditional and contemporary Persian adobe bricks as well as mud–straw plasters, subjecting them to rigorous analysis using a range of techniques aligned with established standards. The subsequent sections delineate the findings obtained through ion chromatography, XRF, XRD, sorption isotherm analysis, and TGA-DSC measurements.

### 4.1. Ion Chromatography Results

The ion chromatography test results for adobe and mud–straw samples are presented in Table 5 and Table 6. The conductivity of the eluates on the adobe samples ranges from 358 µS/cm for the adobe sample C1, made in 1870, to 467 µS/cm for the adobe sample A4, made in 2017. It is 393 µS/cm for adobe sample D2 made in 1320.

The conductivity values for mud–straw samples are significantly higher than those of the adobe samples, with readings of 635 µS/cm for the mud–straw sample X1, produced in 2009, and 1622 µS/cm for the mud–straw sample W3, manufactured in 2019. This notable difference in conductivity could be attributed to the higher straw content in the mud–straw samples (11 wt% for W3 and 8 wt% for X1), contrasting with the adobe samples, which have considerably lower straw content (0.58 wt% for A4, 0 wt% for C1, and 0.02 wt% for D2).

The chloride content in the newly produced mud–straw W3 (2019) is measured at 0.81 wt%. In comparison, the adobe samples A4 (2017) and D2 (1320), originating from the same location, exhibit chloride contents of 0.13 and 0.14 wt%, respectively. Meanwhile, the adobe sample C1 (1870) and mud–straw sample X1 (2009), both sourced from the same area although in different locations, show chloride contents of 0.07 and 0.06 wt%, respectively. The chloride content of the mud–straw W3 is 13.5 times the mud–straw X1, 6.2 times the adobe sample A4, 11.6 times the adobe sample C1, and 5.8 times the adobe sample D2.

It is observed that the conductivity of the new adobe sample A4 is higher than that of the historical adobe samples C1 and D2. Additionally, both mud–straw samples W3 and X1 that are newly produced have high conductivity values. The very high conductivity of sample W3 is caused by a high chloride content of 0.81 wt%. The reason for this can be attributed to the high admixture of straw (11 wt% for sample W3 compared to 8 wt% for sample X1) and the usage of fresh materials in this sample (produced in 2019, contrasting with sample X1 produced in 2009).

According to Wild [62], wheat straw can contain about 0.23 wt% of water-soluble chloride in the plant mass, while rice straw can contain even about 0.58 wt%. On the other hand, the fresh loam or soil used may also have higher chloride concentrations due to the characteristics of the arid climate of the region. Leaching effects due to precipitation after ripening or after harvesting can lead to the leaching of chlorides in wheat straw.

In a similar manner, natural weathering over the years causes the leaching of chloride from the mud-straw plaster and adobe, as observed in sample X1, which has been weathered since 2009.

Although straw has high soluble potassium content and only low concentrations of soluble sodium in the plant mass, the eluates are sodium-accentuated. This suggests that the possible source for the high chloride concentration is the common salt existing in the clay and soil.

### 4.2. XRF and XRD Tests

In order to compare the XRF and XRD test results of the samples in this study with those of other researchers, the results of a series of adobe samples studied by other researchers were collected. The XRD test was carried out for materials under three different moisture conditions: normal (ambient) moisture, completely dry, and saturated. The rationale behind conducting the XRD test at various moisture contents is to identify different minerals associated with distinct moisture levels. This approach allows for the detection of a wider range of minerals that might be present in the materials under different moisture conditions. To make the materials dry, they were exposed to silica gel in a sealed container for four months, during which time they underwent periodic weighing, and they were deemed dry when their weight remained unchanged. To make them saturated, the samples were exposed to humidity in a humidity chamber for four months. To confirm saturation, the samples underwent periodic weighing until their weight showed no further change.

In Table 7, samples from other researchers are coded as follows: M1 (for sample M1, six specimens, i.e., M1(1) to M1(6), were tested, as indicated in Table 8), adobe made in 2016 and used for recent restorations in the town of Ardakan, 60 km from Yazd and 5 km from the town of Maybod [42]; M2, adobe made in 1900 in Jarquyeh, 270 km east of Yazd [43]; M3, adobe made in 2019 and used for recent restoration in Yazd [43]; M4 and M5, adobes made in 1300 in Belqeis Castle in Esfarayen, North-East Iran [40]; M6 and M7, adobes made in 2016 and used for recent restorations in Belqeis Castle [40]; and M8 and M9, adobes made in 2016 and used for recent restorations in the Choga Zambil in Susa, South-East Iran [41]. Figure 3a shows the locations of adobes selected by other researchers.

#### 4.2.1. XRF Results

The XRF test results obtained for adobe and mud–straw samples in this study and adobe samples by other researchers are presented in Table 8 and Table 9.

For adobe and mud–straw samples in this study taken from Maybod, 55 km from Yazd, the dominant chemical compounds are SiO_2_ (about 45 wt%), CaO (around 16 wt%), and Al_2_O_3_ (about 10 wt%). Notably, Ba, Sr, and Zr are prevailing common chemicals found in all samples, with concentrations of approximately 679 ppm, 563 ppm, and 222 ppm, respectively.

The historical and new adobe and mud–straw samples investigated in this study exhibit a relatively similar chemical composition of elements. The locations of the different samples are only a few kilometres apart.

There is a meaningful relationship between the values in Table 4 and Table 8. The percentage of elements in adobe bricks and mud–straw (Table 8) is relatively similar to the percentage of elements in two geological units lava (igneous) and tuff present in the region (Table 4). Note that Table 8 includes the percentage of loss on ignition in the adobe and mud-straw samples, revealing a notable presence of calcium oxide (CaO) within them. Figure 6 shows limestone rocks at the higher altitudes of the region. Since calcium oxide has a high solubility, the presence of a significant percentage of CaO in the adobe and mud–straw samples of the Maybod area is probably due to the dissolved CaO deposited in the soil. If the loss of ignition value is subtracted from Table 8, the CaO value of this table is changed to about 3%, and the composition of the remaining oxide elements is calculated from 100%, the results are similar to Table 4, which indicates a similar chemical composition between lava and tuff rocks of the region and the soil composition used in the adobe bricks and mud–straw of Maybod.

Specimens of the adobe sample M1 [42] taken from Ardakan, 5 km from Maybod and 60 km from Yazd, have a similar chemical composition. As mentioned before, six specimens, i.e., M1(1) to M1(6), were tested for sample M1. The dominant compounds are SiO_2_ with about 42 wt%, CaO with around 15 wt%, Al_2_O_3_ with about 10 wt%, and MgO with 6 wt%. The amount of loss of ignition is also comparable. The similarity of the samples in Ardakan and Maybod is due to comparable soil, as these two towns are only 5 km away.

In the adobe sample M9 [41], the order of abundance of chemical elements is the same, dominated by SiO_2_ with 33 wt%, CaO with 22 wt%, Al_2_O_3_ with 8 wt%, and MgO with 6 wt%. The loss of ignition, on the other hand, is significantly higher, and the high CaO content of the sample suggests a higher carbonate content. This new adobe sample was produced in Susa, about 900 km from Maybod.

#### 4.2.2. XRD Results

**Figure 7:**
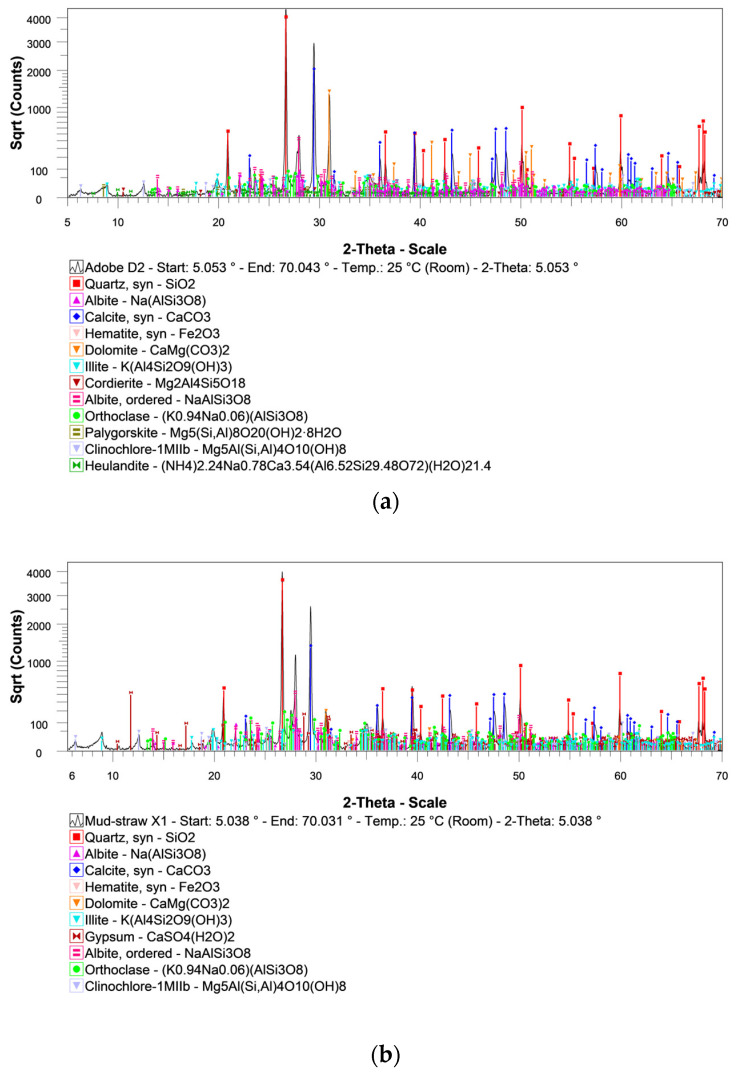
XRD pattern for materials at normal (ambient) moisture: (**a**) adobe D2, (**b**) mud–straw X1.

As an example of the XRD patterns obtained for the adobe and mud–straw samples in the present study, the XRD patterns for the adobe sample D2 and the mud–straw sample X1 at normal (ambient) moisture are illustrated in Figure 7. Mineralogical compositions identified by XRD for the adobe and mud–straw samples at normal moisture in this study and adobe samples by other researchers are presented in Table 10.

In general, the majority of compounds found in the adobe and mud–straw samples studied in this research are similar. The common compounds in all samples include quartz (SiO_2_), and different feldspars such as albite (Na(AlSi_3_O_8_)) and orthoclase ((K_0.94_Na_0.06_)(AlSi_3_O_8_)). Furthermore, calcite (CaCO_3_), dolomite (CaMg(CO_3_)_2_) and different clay minerals, clinochlore-1Mllb (Mg_5_Al(Si, Al)_4_O_10_), and illite (K(Al_4_Si_2_O_9_(OH)_3_)) are present in all samples. Hematite (Fe_2_O_3_) also exists in the samples except in the adobe sample C1, made in 1870; the mud–straw sample W3, made in 2019; and the mud–straw sample X1.

In the historical adobe samples, C1 made in 1870 and D2 made in 1320, additional compounds exist. Palygorskite (Mg_5_(SiAl)_8_O_20_(OH)_2.8_) is observed in both the adobe samples C1 and D2. The adobe sample C1 contains ferroactinolite ((Ca,Na,K)_2_Fe_5_Si_8_O_22_), while D2 contains cordierite (Mg_2_Al_4_Si_5_O_18_) and heulandite ((X)_3_(Al_3_Si_9_O_24_)*7-8H_2_O).

In all studied samples, swelling clay minerals, like montmorillonite and smectite, are present in very small amounts, while the amount of non-swelling clay mineral, illite, is significant. This low presence of swelling clay minerals is important for producing good quality adobe bricks, as they tend to cause fissures during drying if they contain high amounts of swelling clay minerals. The absence of significant amounts of swelling clay minerals in the samples is a positive indicator for adobe brick production. This means that the risk of deformation and cracking during the drying process is significantly reduced. Additionally, the presence of quartz is consistent with the typical composition of desert soils in the region and aligns with the geological units of the area.

The adobe sample M2 [43], made in 1900 in Jarquyeh, 270 km east of Yazd, and the adobe sample M3 [43], made in 2019 in Yazd, show the greatest similarity in mineral content. They contain quartz (SiO_2_), albite (NaAlSi_3_O_8_), calcite (CaCO_3_), clinochlore-1Mllb (Mg_5_Al(SiAl)_4_O_10_), and dolomite (CaMg(CO_3_)_2_). Other adobe samples from other locations have a completely different mineralogical composition; the reason is the different types of soils at different locations far from each other.

The XRD results show that quartz (SiO_2_) and calcite (CaCO_3_) are present in all the adobe and mud–straw samples shown in Table 10 except the adobe sample M5.

In addition, the XRF results in Table 8 show a comparable chemical composition to the adobe sample of type M1 from Dormohamadi and Rahimnia [42]. Adobe samples of the series M1 in Table 8 are made in Ardakan with the same soil used for adobe samples studied in this research, while the M9 adobe sample, with significantly lower concentrations of SiO_2_ and Al_2_O_3_ but higher contents of CaO, was made in Susa, very far from Ardakan.

Upon comparing the XRD patterns of completely dry and saturated samples, it becomes evident that the three adobe samples contain the same minerals; however, the varying heights of the peaks indicate differences in the mineral quantities among the different adobe samples. The comparison between the mud–straw samples reveals the presence of gypsum in the mud–straw sample X1, while the mud–straw sample W3 lacks gypsum.

Examining all adobe and mud–straw samples reveals that they possess similar minerals but differ in their respective amounts. Interestingly, only the mud–straw sample X1 contains gypsum; the other samples do not.

The presence of gypsum in the mud–straw sample X1 may be attributed to various factors, including variations in the composition of raw materials used during the construction process. It is most likely that gypsum, a naturally occurring mineral, was unintentionally added to the materials used for constructing the mud–straw sample X1. One reasonable explanation could be the accidental incorporation of gypsum from other applications within the adobe building. Gypsum is commonly used in various construction practices in adobe buildings, such as creating gypsum forms for constructing arches and vaults. These forms provide structural support and shape for adobe arches and vaults during construction. Additionally, gypsum mortar is frequently used to fill voids in mortar joints to enhance the strength and integrity of adobe vaults. It is conceivable that gypsum from these forms and mortar applications might have unintentionally been mixed with the components of the mud–straw plaster during construction. Furthermore, both the mud–straw samples X1 and W3 were used for plastering the same building. Sample X1, containing gypsum, was produced and applied to the building in 2009, while sample W3, containing no gypsum, was produced and applied to the building in 2019. Both samples were produced from the same soil using the same production technique. This similarity in production methods and materials between samples X1 and W3 further supports the notion that the presence of gypsum in sample X1 is likely accidental, possibly due to the use of gypsum for other applications within the construction process.

### 4.3. Sorption Isotherm Results

The determination of moisture sorption isotherms was conducted for two sets of samples. The first set comprised three types of adobe samples of A4, D2, and C1, along with two types of mud–straw samples of W3 and X1, for comparison. Straw was consistently present in the mud–straw samples in this set, reflecting real-world mud–straw plaster.

The second set exclusively consisted of the mud–straw samples W3 and X1 examined in two scenarios: with and without the addition of straw. This examination aimed to assess the influence of straw on the sorption isotherm characteristics of mud–straw samples.

#### 4.3.1. Comparative Analysis of Adobe and Mud–Straw Samples

**Figure 8:**
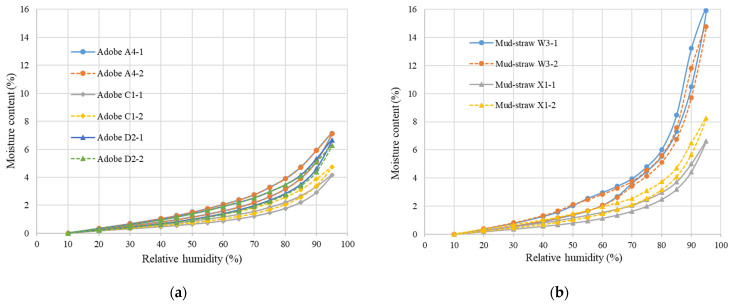
Sorption isotherm and desorption curves for test duration of 30 days: (**a**) adobe, (**b**) mud–straw.

**Figure 9:**
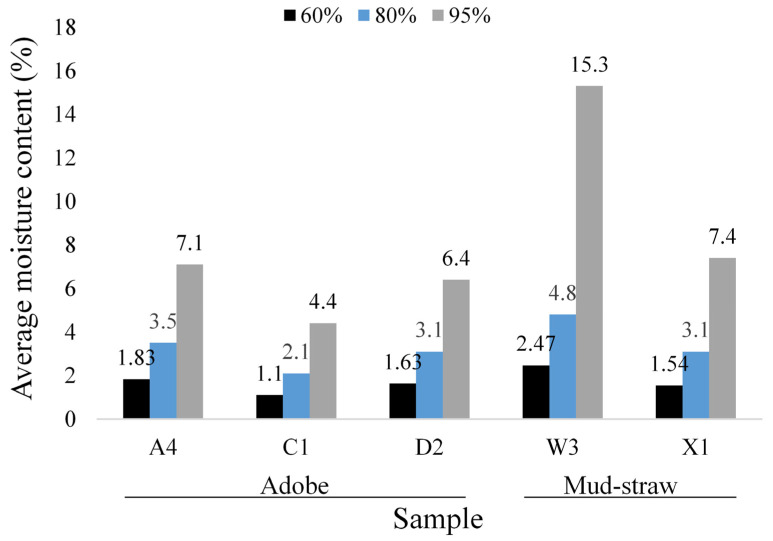
Average moisture content of adobe and mud–straw (containing straw) samples at relative humidities of 60%, 80%, and 95% for test duration of 30 days.

Duplicate determinations were conducted for each type of adobe and mud–straw plaster over a period of 30 days. Sorption isotherm (the lower) and desorption (the upper) curves of adobe and mud–straw samples in this study for the test duration of 30 days are shown in Figure 8. Table 11 and Figure 9 present the average moisture contents at the relative humidities of 60%, 80%, and 95%. The moisture content, called the equilibrium moisture content (EMC), at a relative humidity of 80% (EMC_80_), is an important characteristic variable for physical simulations [1]. The moisture content at a relative humidity of 95% (EMC_95_) is used for comparison with other researchers’ findings. EMC_60_, chosen for comparison, enriches the study by examining the materials’ response to lower humidity, complementing the exploration of varied moisture environments alongside EMC_80_ and EMC_95_.

Among the adobe samples, adobe C1, made in 1870, exhibits the lowest moisture content and adobe A4, made in 2017, shows the highest moisture content. This is in relation to the amount of straw in the adobe samples, where adobe C1 has the lowest straw content of 0 wt%, and adobe A4 has the highest straw content of 0.58%. At the relative humidity of 60%, the moisture content (EMC_60_) for adobe samples A4, C1, and D2 are, respectively, 1.83%, 1.1%, and 1.63%. The values for the relative humidity of 80% (EMC_80_) are 3.5%, 2.1%, and 3.1%, respectively. The values are, respectively, 7.1%, 4.4%, and 6.4% for the relative humidity of 95% (EMC_95_). Other researchers have reported equilibrium moisture content equal to 0.5% to 7% [63], 4% to 6% [64], 3.5% [12], 5.3% [65], 3% to 5% [18], and 1.3% [20].

In the case of the mud–straw samples, mud–straw W3, produced in 2019, exhibits the highest moisture content, while mud–straw X1, produced in 2009, displays the lowest moisture content. There is once more a correlation between the moisture content and the straw amount, with mud–straw W3 containing a higher straw content of 11 wt% compared to mud–straw X1, which contains a lower straw content of 8 wt%. The moisture content at the relative humidity of 60% (EMC_60_) for sample W3 is 2.5% and that of sample X1 is 0.59%. These values at the relative humidity of 80% (EMC_80_) are, respectively, 4.8% and 3.1%. At the relative humidity of 95% (EMC_95_), these values are 15.3% for sample W3 and 7.4% for sample X1. Ashour et al. [14] reported an equilibrium moisture content of less than 7% for mud–straw plaster with wheat straw, barley straw, and wood shavings.

**Figure 10:**
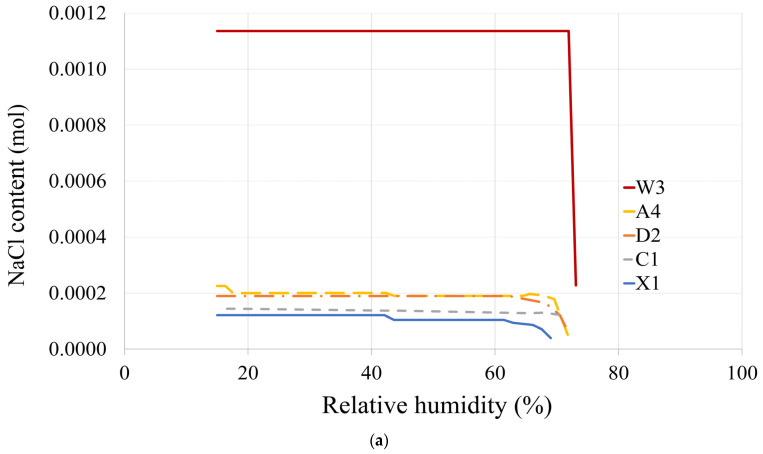

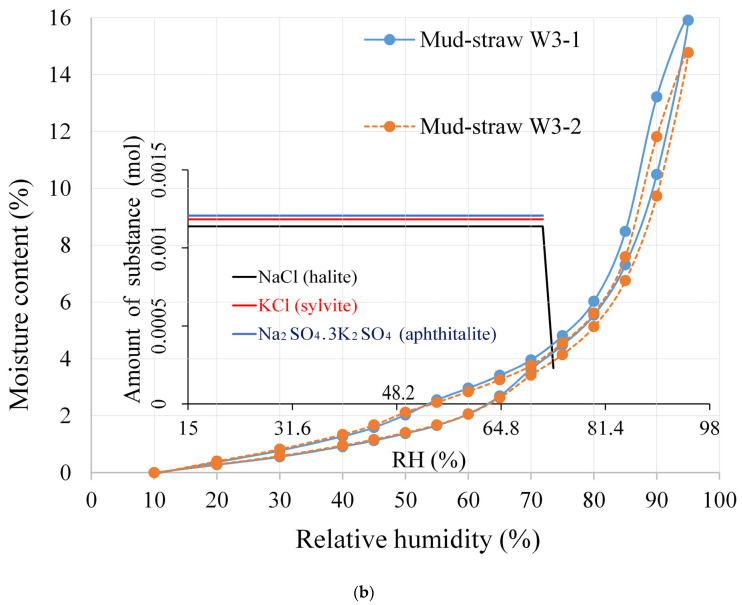
(**a**) Variation in amount of common salt content versus relative humidity for adobe and mud–straw samples, (**b**) variation in amount of salt mixtures versus relative humidity for mud–straw W3 (containing straw) together with sorption isotherm and desorption curves (test duration of 30 days).

It is observed that adobe A4, D2, and the mud–straw sample W3, which are more heavily loaded with common salt (Table 5, Table 6 and Table 11), also have significantly higher moisture absorption. The analysis of the variation in the amount of salt mixtures with respect to relative humidity for samples was conducted using RUNSALT 1.9 software [66]. Figure 10a displays the variation in the amount of common salt content, NaCl, versus relative humidity for the adobe and mud–straw samples. Table 11 indicates that the amount of common salt in the mud–straw sample W3 (11.36 × 10^−4^ mol) is 9.3 times the mud–straw sample X1, 5 times the adobe sample A4, 8 times the adobe sample C1, and 5.9 times the adobe sample D2. The analysis of the variation in the amount of salt mixtures with respect to relative humidity for the mud–straw sample W3 is depicted alongside the sorption isotherms and desorption curves in Figure 10b. It can be seen that at the relative humidity of about 71%, all salt is dissolved, and from then on, the second curve shows a sudden increase in slope, which means a significant increase in moisture absorption.

#### 4.3.2. Influence of Straw on Mud–Straw Sorption Isotherms

Two additional sets of sorption isotherm tests were conducted to study the effect of the presence of straw in the mud–straw samples. Tests were performed once on mud–straw samples with straw and once without straw. The tests were conducted for 60 days, which simulated long-term moisture exposure.

**Figure 11:**
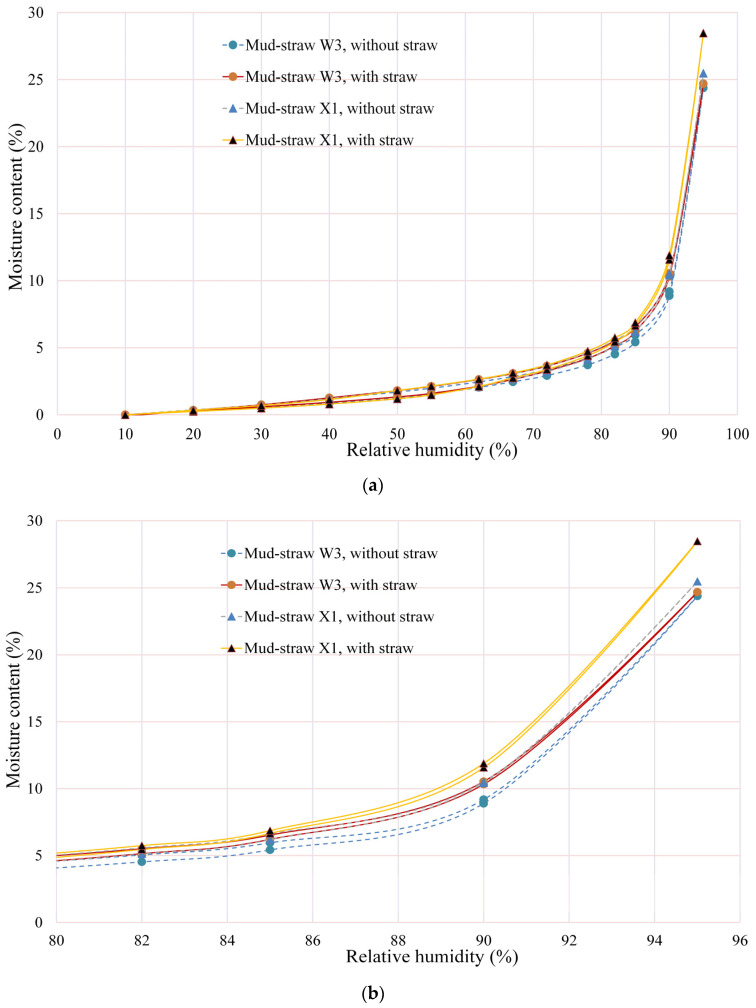
(**a**) Sorption isotherm and desorption curves for mud–straw samples with and without straw for a test duration of 60 days, (**b**) inset of the curve at high relative humidity.

Figure 11 shows the sorption isotherm and desorption curves of the mud–straw samples with and without straw for the test duration of 60 days. The presence of straw in the mud–straw samples leads to higher moisture content at all relative humidities compared to the samples without straw.

### 4.4. TGA and DSC Results

**Figure 12:**
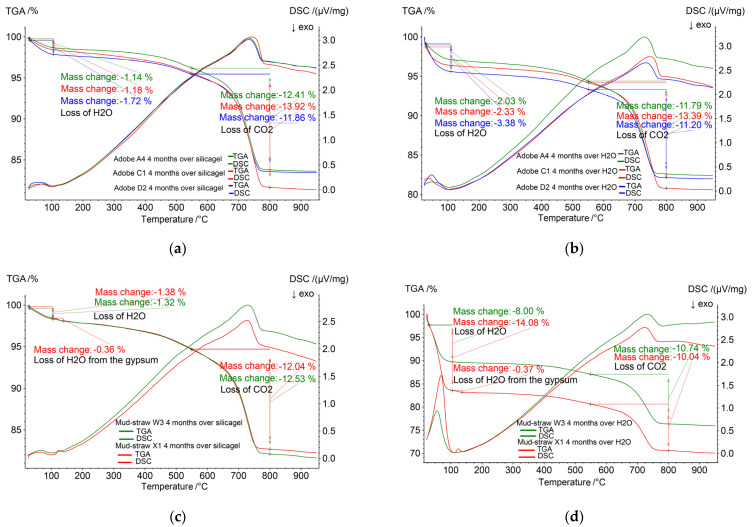
TGA and DSC curves: (**a**) completely dry adobe samples, (**b**) saturated adobe samples, (**c**) completely dry mud–straw samples, (**d**) saturated mud–straw samples.

The results of thermogravimetric analysis and differential scanning calorimetry for the adobe and mud–straw samples are illustrated in Figure 12 and summarised in Table 12. In the completely dry adobe samples A4, C1, and D2, water loss occurs between 30 °C and 105 °C, with mass losses of 1.14%, 1.18%, and 1.72%, respectively. For saturated adobe samples, these values increase to 2.03%, 2.33%, and 3.38% for samples A4, C1, and D2, respectively. Major carbon dioxide loss is observed between 550 °C and 800 °C, with mass losses of 12.41%, 13.91%, and 11.86% for the dry adobe samples A4, C1, and D2, respectively. For saturated samples, these values are 11.79%, 13.39%, and 11.2%.

In both the completely dry and saturated adobe samples, at temperatures between 30 °C and 105 °C, heating causes the release of water (H_2_O) through the process of dehydration. This is also observable as a peak in the corresponding DSC curve between 30 °C and 105 °C. On the other hand, at temperatures between 550 °C and 800 °C, the samples undergo decarboxylation due to heating, and hence, carbon dioxide (CO_2_) is released. The mass loss due to decarboxylation is observed at a peak in the corresponding DSC curve between 550 °C and 800 °C.

A similar trend holds for the completely dry and saturated mud–straw samples. At temperatures ranging from 30 °C to 105 °C, loss of water is observed. In the dry samples, water loss is 1.32% for sample W3 and 1.38% for sample X1, and in saturated samples, it is 8% for sample W3 and 14.08% for sample X1, which corresponds to the endothermic peaks of the DSC curves. In the mud–straw sample X1, an abrupt peak is observed at temperatures from 105 °C to 136 °C. This peak can be attributed to the loss of excess water from gypsum present in this sample, as shown in Figure 12c,d. The gypsum in the mud–straw sample X1 is traced in Figure 7b. Also, at temperatures ranging from 550 °C to 800 °C, loss of carbon dioxide occurs with values of 12.04% and 12.53% of the completely dry X1 and W3 adobe samples, respectively, and 10.04% of the saturated adobe sample X1 and 10.74% of the saturated adobe sample W3, corresponding to the peaks of the DSC curves between 550 °C and 800 °C (Figure 12c,d).

At low temperatures from 30 °C to 105 °C, for the mud–straw samples, which are without straw content in the TGA and DSC tests, under both completely dry and saturated conditions, the water loss of the mud–straw sample X1 is more than that of the mud–straw sample W3. This corresponds to the characteristics of their sorption isotherm and desorption curves shown in Figure 11 and the values presented in Table 12, in which the moisture content of the X1 sample without straw is higher than the W3 sample without straw. The mud–straw sample X1, with a higher moisture content, loses more water than the mud–straw sample W3, with a lower moisture content.

In both the adobe and mud–straw samples, as indicated in Table 10, the tested samples include calcite (CaCO_3_). At high temperatures from 550 °C to 800 °C, the calcite is decomposed to calcium oxide (CaO) and carbon dioxide (CO_2_), and the samples release CO_2_ (Figure 12). This process leads to mass loss due to decarboxylation.

Table 8 presents the XRF analysis results, further detailed in Table 12, revealing significant variations in the CaO content within the adobe group. Among these samples, the adobe sample C1 exhibits the highest CaO content at 17.92 wt%, while the adobe sample A4 displays the lowest CaO content at 15.82 wt%. These differences suggest potential differences in the CaCO_3_ content, with C1 potentially containing a higher proportion of CaCO_3_ compared to A4. This correspondence aligns with the observed trend in CO_2_ loss from the TGA and DSC results. Under completely dry conditions, sample C1 displays a CO_2_ loss of 13.91%, whereas sample A4 exhibits 12.41%. Similarly, under saturated conditions, the CO_2_ loss is 13.39% for C1 and 11.79% for A4.

Similar trends are observed within the mud–straw group, where the W3 sample demonstrates a higher CaO content at 17.35 wt% compared to the X1 sample, which exhibits a lower content of 15.14 wt%. Correspondingly, the CO_2_ loss trend follows a similar pattern, with the W3 sample exhibiting higher CO_2_ loss percentages compared to the X1 sample. Under completely dry conditions, the W3 sample shows a CO_2_ loss of 12.53%, whereas the X1 sample exhibits 12.04%. Similarly, under saturated conditions, the CO_2_ loss is 10.74% for W3 and 10.04% for X1.

## 5. Discussion on Results

The results obtained from XRF analysis indicate that a similar chemical composition exists in all studied adobe and mud–straw samples, both historical and new. The chemical elements with higher amounts are, respectively, Ba at about 679 ppm, then Sr (563 ppm) and Zr (222 ppm). The dominant chemical compounds are SiO_2_, with about 45 wt%, followed by CaO (17 wt%) and Al_2_O_3_ (10 wt%). The similarity of chemical elements and compounds in the samples could be related to the same type of soil in this area.

The XRD results indicate that all samples studied have a similar mineralogical composition. The common phases in all samples are quartz, feldspars (albite and orthoclase), calcite, dolomite, and clay minerals (clinochlore and illite). Other phases are also observed in some samples. Clay minerals with high swelling potential, like montmorillonite and smectite, are absent in the studied samples, whereas the non-swelling clay mineral illite is present.

The ion chromatography results show that the mud–straw samples have higher conductivity values than the adobe samples. This can be attributed to the presence of straw in the mud–straw samples.

It is observed that the conductivity of new adobe and mud–straw samples is higher than historical ones. The very high conductivity of one of the mud–straw samples is caused by a high chloride content. The reason for this could be the high admixture of straw and the freshness of the material used in this sample. On the other hand, the fresh loam or soil used may also have higher chloride concentrations caused by the arid climate and soil salinisation. Likewise, natural weathering of the mud–straw plaster and the adobe over the years might have led to the leaching of chloride.

The results obtained from isotherm sorption determination show that adobe and mud–straw plaster with considerably higher moisture absorption are those with a higher load of chlorides. Adobe samples with higher straw contents exhibit higher moisture content.

The thermogravimetric analysis and differential scanning calorimetry results indicate that at temperatures between 100 °C and 125 °C, the loss of water of the completely dry and saturated adobe samples varies from 0.61% to 1.72% and from 2.03% to 3.38%, respectively, whereas that of the completely and saturated mud–straw samples ranges from 1.32% to 1.38% and from 8% to 14.08%, respectively. At temperatures between 650 °C and 800 °C, the range of the loss of carbon dioxide of the completely dry and saturated adobe samples is, respectively, between 11.86% and 14.4% and between 11.2% and 13.39%, while that of the completely dry and saturated mud–straw samples ranges from 12.04% to 12.53% and from 10.04% to 10.74%.

## 6. Implications for Restoration and Rehabilitation Practices

Preserving the architectural heritage of adobe structures requires a thorough understanding of their material characteristics and structural behaviour. By adhering to the principles outlined by the ICOMOS-ISCARSAH Committee [3], restoration and rehabilitation practices can effectively address the challenges posed by ageing and environmental factors. This section briefly explores the implications of the study findings on intervention solutions, the compatibility of material properties, and considerations for restoration and rehabilitation approaches for Persian adobe buildings.

### 6.1. Influence of Sample Specificity on Intervention Solutions

The specificity of each sample plays a crucial role in determining appropriate intervention solutions for restoration and rehabilitation projects. As recommended by the ICOMOS-ISCARSAH Committee, a comprehensive understanding of the structural behaviour and material characteristics is essential [3]. Variations in elemental composition, mineralogical composition, and moisture sorption behaviour between samples can influence the selection of conservation treatments and restoration techniques. For instance, insights gained from XRF and XRD analyses provide valuable information about the elemental and mineralogical composition of adobe and mud–straw plaster, guiding decisions on suitable consolidation methods and protective coatings to enhance structural integrity and durability over time. Furthermore, ion chromatography reveals the presence of soluble salts and ions within these materials, informing strategies to mitigate moisture-related damage and ensure long-term stability and compatibility with restoration interventions.

### 6.2. Compatibility of Adobe and Mud–Straw Plaster Properties

The compatibility of new materials, such as adobe and mud–straw plaster, with the original materials used in restoration work is paramount, as emphasised in the ICOMOS-ISCARSAH recommendations [3]. Understanding the elemental composition, mineralogical characteristics, and moisture sorption behaviour of adobe and mud–straw plaster is crucial for assessing their compatibility with existing materials and identifying potential risks of incompatibility. It is imperative that the materials used in restoration interventions complement the original construction materials to ensure structural integrity and authenticity in heritage conservation efforts.

In the case of adobe buildings, the compatibility of adobe with mud–straw plaster, which serves as a protective coating, is particularly important. Adobe is vulnerable to moisture-related damage, such as efflorescence or salt crystallisation, which can compromise its structural stability over time. Mud–straw plaster acts as a barrier against environmental elements, providing insulation and protecting the adobe from moisture ingress. Therefore, ensuring compatibility between adobe and mud–straw plaster is essential to maintain the structural integrity and longevity of adobe structures.

Furthermore, TGA-DSC analyses provide insights into the thermal decomposition behaviour of these materials, informing decisions related to fire safety measures and the selection of appropriate fire-retardant treatments during restoration efforts. Additionally, TGA-DSC analyses help identify any organic and inorganic additives present in the materials, influencing their structural integrity, durability, and susceptibility to degradation over time.

### 6.3. Considerations for Rehabilitation Solutions

In the domain of restoration and rehabilitation practices, an important consideration is the compatibility of materials, ensuring the integrity, authenticity, and longevity of heritage structures. This paper highlights the fundamental significance of material compatibility by providing comprehensive insights into the elemental composition, mineralogical characteristics, and thermal characteristics of adobe and mud–straw plaster used in Persian adobe buildings.

Several restoration and rehabilitation approaches can be employed to address the conservation needs of Persian adobe buildings. Drawing inspiration from established preservation practices and innovative restoration techniques, restoration practitioners can develop tailored solutions to enhance structural stability, mitigate decay risks, and preserve architectural authenticity. By incorporating sustainable building practices and modern conservation technologies, rehabilitation projects can achieve a balance between preserving heritage values and meeting contemporary standards of safety and comfort.

In all restoration and rehabilitation activities, the compatibility of materials used is essential. The selection of materials must align with the original construction methods and existing fabric of the structure to ensure harmonious integration and long-term preservation. Examples of rehabilitation solutions include consolidation treatments, protective coatings, and adaptive reuse strategies, each carefully selected to address the unique challenges posed by Persian adobe architecture.

## 7. Conclusions

In this study, ion chromatography, XRF analysis, XRD analysis, moisture sorption isotherm determination, thermogravimetric analysis, and differential scanning calorimetry of a number of historical and new adobe and mud–straw samples were conducted. Samples were taken from historical buildings, and new adobe bricks and mud–straw plaster were used in the same places for restoration, except for one mud–straw sample that was made in the laboratory using materials from the site. The selected historical buildings are a few kilometres apart, all located in the city of Maybod, 55 km east of the city of Yazd, Central Iran.

The comprehensive analysis conducted on the adobe and mud–straw samples has provided valuable insights into their elemental composition, mineralogical characteristics, moisture sorption behaviours, and thermal properties. The consistent chemical compositions, predominantly featuring SiO_2_, CaO, and Al_2_O_3_, reflect the prevalent soil types in the studied region. This uniformity signifies a common geological origin, establishing a foundational understanding of construction materials within this region.

The mineralogical investigation identified key compounds, like quartz, feldspars (albite and orthoclase), calcite, dolomite, and clay minerals (clinochlore and illite), across all samples. The absence of high-swelling clay minerals, often associated with structural weaknesses during the drying process, indicates the suitability of these materials for adobe brick production.

The study of moisture sorption highlighted the impact of material composition on hygroscopic behaviour. The presence of straw notably increased moisture retention, which was especially evident in the mud–straw samples.

Moreover, the thermal analyses reveal distinctive dehydration and decomposition processes in both the adobe and mud–straw samples. The observed release patterns of water and carbon dioxide provided insights into the materials’ thermal responses and chemical decomposition mechanisms.

Additionally, this study addressed a notable gap in the existing literature concerning the comprehensive characterisation of Persian adobe and mud–straw plaster. By extending the scope of analysis to include ion chromatography, moisture sorption isotherm determination, and thermogravimetric analysis with differential scanning calorimetry, this research contributed to filling this gap and advancing the knowledge base in the field of traditional building materials. Of particular significance is the lack of previous studies focusing on mud–straw, highlighting the importance of the present research.

These collective findings hold significant implications for construction practices and restoration efforts, offering a deeper understanding of the structural, compositional, and thermal attributes of adobe and mud–straw materials. The insights gleaned from this study provide valuable guidance for construction projects, facilitating informed decisions in material selection, structural design, and restoration methodologies. However, further research and experimentation in varied environmental conditions is needed to enhance this understanding and refine the application and preservation strategies of these traditional building materials in contemporary construction practices.

## Figures and Tables

**Table 1:** Quantities of clay soil, adobe brick, and mud–straw plaster components [7].

Material	Clay Soil Components		Adobe Brick and Mud–Straw Plaster Components		
	Clay Content (wt%)	Earth Content (wt%)	Clay Soil Content (wt%)	Straw Content (wt%)	Water to Clay Soil Ratio (%)
Adobe brick	30–40	60–70	99.5	0.5	25
Mud–straw plaster	30–40	60–70	90–95	5–10	25

**Table 2:** Summary of key findings from literature review on adobe and clay-based plasters.

Researcher	Focus	Key Findings
Coffman et al. [8]	Mineralogical study of adobe bricks from historical buildings worldwide	Determined mineral composition and grain-size distribution of adobe bricks; found consolidation effectiveness dependent on clay mineralogy and grain size.
Baglioni et al. [9]	Properties of rammed earth and adobe in Morocco	Found higher clay content in adobe compared to rammed earth; identified mineral composition, including quartz, feldspars, and expandable clay minerals; concluded old adobe more suitable for construction due to higher clay content.
Fratini et al. [10]	Properties of adobe in Italy	Identified mineral composition including quartz, feldspars, and clay minerals; observed variations in clay mineral types; noted significance of calcite in some samples.
Costa et al. [11]	Influence of mineral composition on adobe properties in Portugal	Found kaolinite, illite, and smectite as main clay minerals; noted positive effect of phyllosilicate content on absorption/drying and mechanical properties of adobe bricks.
El Fgaier et al. [12]	Hygroscopic properties of unfired clay bricks in France	Showed higher sorption capacity compared to fired clay bricks; observed moisture content increase with relative humidity; highlighted potential for indoor climate regulation.
De Castrillo et al. [13]	Comparison of adobe bricks across historical periods in Cyprus	Identified dominant minerals, including calcite, quartz, and albite; noted variations in chemical composition attributed to regional soil differences.
Ashour et al. [14]	Equilibrium moisture content of mud–straw plaster in unspecified location	Observed increase in equilibrium moisture content with humidity and decrease with temperature; found relative humidity has more pronounced effect than temperature.
Costa et al. [15]	Mineralogical characterisation of adobe in central Portugal	Identified four groups of adobe based on mineral composition; quartz, calcite, and phyllosilicates predominant; variations in iron-oxide content noted.
Sanchez-Calvillo et al. [16]	Characterisation of adobe from Jojutla de Juarez, Mexico	Identified calcite and kaolinite as major minerals; noted low swelling and shrinkage properties of kaolinite.
Laborel-Préneron et al. [17]	Hygrothermal properties of earthen materials with plant aggregates	Showed low thermal conductivity with a large volume of plant aggregates; minimal improvement in water vapour permeability observed.
Gomes [18]	Hygrothermal characteristics of earthen materials in unspecified location	Determined sorption and desorption curves; observed maximum water content of 3–5%.
Mellaikhafi et al. [19]	Physical, chemical, and geotechnical tests of soils for rammed earth and adobe in Morocco	Identified quartz, calcite, and ferroan clinochlore as main minerals; absent kaolinite and illite; observed absence of swelling minerals, such as smectite and vermiculite.
Ranesi et al. [20]	Relative humidity properties of plastering mortars in unspecified location	Identified earth plaster as most suitable for humidity regulation; gypsum–air lime combination found more effective than pure gypsum.
Lima et al. [21]	Effect of clay mineralogy on earth plaster properties in unspecified location	Found significant impact of clay mineralogy on plaster properties, including vapour adsorption, drying shrinkage, and mechanical strength.
Savadogo et al. [22]	Physico-mechanical properties of earthen plaster stabilised with fermented rice husk	Observed improvements in properties with addition of rice husk; noted negative impact on water absorption and erosion resistance with excessive rice-husk content.
Santos et al. [23]	Efficiency of earth plaster on various masonry types	Demonstrated durability of earth plaster across different substrates; exhibited long-term stability.
Vares et al. [24]	Hygrothermal performance of clay–sand plaster in unspecified location	Evaluated moisture buffering and vapour-permeability properties; emphasised importance of material selection for indoor climate control.
Santos and Faria [25]	Characterisation of earthen plasters in unspecified location	Evaluated mechanical properties and durability of various mortar formulations; noted influence of additives on plaster performance.
Santos et al. [26]	Comparative analysis of mineralogical and hygroscopic properties of plasters in unspecified location	Examined mechanical and hygroscopic properties of different plasters; noted lower mechanical strength but higher hygroscopicity in earthen plasters.
Ojo et al. [27]	Characteristics of unfired earthen materials in unspecified location	Investigated characteristics and properties of earthen materials; found alkali activation improved physical and mechanical properties.
Bass et al. [28]	Microstructure and mineral composition of ancient earthen plaster in American Southwest	Studied microstructure, minerals, and deterioration mechanisms; highlighted adaptability of plaster to site conditions.
Muşkara and Bozbaş [29]	Archaeometric investigations of earthen building materials in Turkey	Analysed mineralogical and chemical properties of earthen materials from vernacular houses; aimed to understand building technologies and raw material properties for restoration strategies.
Saleh [30]	Analysis of natural adobe plaster in Jordan Valley	Analysed plaster morphology; found significant variance in plaster recipes across regions.
Saleh [31]	Experimental characterisation of wall plaster in Al-Ulla, Saudi Arabia	Characterised wall plaster using various methods; identified four types of plaster.
Silveira et al. [32]	Characterisation and rehabilitation of adobe constructions in Portugal	Characterised adobe composition, mechanical behaviour, and structural performance; suggested methods for enhancing durability and seismic resistance.
Sánchez et al. [33]	Review of adobe masonry properties for rehabilitation in unspecified location	Compiled data on adobe mechanical properties from various studies; highlighted variations in regulations for local conditions.
Papayianni and Pachta [34]	Analysis and repair of historical earth block masonry in Greece	Developed methodology for analysis and repair of historical earth block houses; tested compatible repair materials based on earth.
González-Sánchez et al. [35]	Investigation of stabilised earthen mixtures for adobe preservation in Mexico	Evaluated eco-friendly earthen mixture for enhanced durability; applied mixtures in adobe dwellings with promising results.
Faria et al. [36]	Experimental characterisation of earth plastering mortar for adobe rehabilitation	Conducted tests on earth plastering mortar; found it suitable for rehabilitation of historical adobe buildings.
Gomes et al. [37]	Compatibility of earth-based repair mortars with rammed earth substrates	Tested repair mortars on rammed earth blocks; observed variations in mortar behaviour based on support type.
Jia et al. [38]	Analysis of historical earthen plaster for restoration	Investigated plaster properties; found certain additives improved properties, while others led to cracking.
Mattone et al. [39]	Testing of earth–gypsum plasters for conservation of earthen constructions	Developed plasters to protect earthen heritage; tested mixtures with natural and synthetic additives.
Hosseini et al. [40]	Mineralogical and physical tests on historical and new adobe in Iran	Determined mineral composition; found predominance of quartz and calcite.
Zakavi [41]	Mineralogical analysis of soil for new adobe in Iran	Identified dominant minerals; noted soil’s poor clay content.
Dormohamadi and Rahimnia [42]	Physical, mineralogical, and mechanical tests of adobe in Iran	Identified main minerals; observed low swelling clay content in soil.
Eskandari [43]	Properties of historical and new adobe bricks in Iran	Identified mineral composition; found quartz as significant constituent.

**Table 3:** Types of adobe and mud–straw studied in the present study.

Material	Code	Location	Period	Comment	Length (cm)	Width (cm)	Thickness (cm)	Corresponding Geology Region	Straw Content (wt%)
Adobe	A4	Narin Castle (Maybod)	2017	Used for recent restorations	21	21	5.4	Cultivated land (Q^cu^ unit)	0.58
	C1	Kazem Abad village (Maybod)	Probably 1870	Original	22.5	23	6	Cultivated land (Q^cu^ unit)	0
	D2	Narin Castle (Maybod)	Probably 1320	Original	20	24.5	5.5	Cultivated land (Q^cu^ unit)	0.02
Mud–straw	W3	Deh-Naw Mosque (Maybod)	2019	Made in the laboratory for the present study from materials used for recent restorations	20	20.5	2.7	Cultivated land (Q^cu^ unit)	11
	X1	Deh-Naw Mosque (Maybod)	2009	Used for previous restorations	24	18	1.5	Cultivated land (Q^cu^ unit)	8

**Table 4:** Chemical composition of lava and tuff unit samples (percentage of weight, wt%) [49].

	SiO_2_	Al_2_O_3_	Fe_2_O_3_	MgO	CaO	Na_2_O	K_2_O	MnO	TiO_2_	P_2_O_5_
Sample 1	65.36	14.77	2.06	2.84	3.17	3.88	4.20	0.01	0.63	0.17
Sample 2	60.75	15.26	8.45	4.68	3.27	2.71	2.29	0.04	0.69	0.23

**Table 5:** Ion chromatography test results for adobe and mud–straw samples: concentration of anions.

Sample	Cl^−^ (wt%)	NO_2_^−^ (wt%)	NO_3_^−^ (wt%)	SO_4_^2−^ (wt%)	Conductivity (µS/cm)
Adobe A4	0.13	<0.01	0.01	0.14	467
Adobe C1	0.07	<0.01	0.01	0.12	358
Adobe D2	0.14	<0.01	0.02	0.06	393
Mud–straw W3	0.81	<0.01	<0.01	0.15	1622
Mud–straw X	0.06	0.01	0.03	0.35	635

**Table 6:** Ion chromatography test results for adobe and mud–straw samples: concentration of cations.

Sample	Na^+^ (wt%)	NH_4_^+^ (wt%)	K^+^ (wt%)	Mg^2+^ (wt%)	Ca^2+^ (wt%)
Adobe A4	0.12	<0.01	0.02	0.01	0.04
Adobe C1	0.09	<0.01	0.01	0.01	0.03
Adobe D2	0.11	<0.01	0.01	0.01	0.02
Mud–straw W3	0.54	<0.01	0.13	0.05	0.10
Mud–straw X1	0.07	0.01	0.03	0.01	0.14

**Table 7:** Types of adobe studied by other researchers.

Material	Code	Location	Period	Comment	Reference
Adobe	M1	Ardakan (60 km east of Yazd)	2016	Used for recent restorations	[42]
	M2	Jarquyeh (270 km east of Yazd)	Probably 1900	Original	[43]
	M3	Yazd	2019	Used for recent restorations	[43]
	M4, M5	Belqeis Castle (Esfarayen, North-East Iran)	Probably 1300	Original	[40]
	M6, M7	Belqeis Castle (Esfarayen, North-East Iran)	2016	Used for recent restorations	[40]
	M8, M9	Choga Zambil (Susa, South-East Iran)	2016	Used for recent restorations	[41]

**Table 8:** Chemical composition analysed by XRF for adobe and mud–straw samples in this study and adobe samples by other researchers (percentage of weight, wt%; main element composition as oxides).

Oxides	This Study					Other Researchers						
	Location											
	Maybod					Ardakan						Susa
	Adobe			Mud–Straw		Adobe						
	A4	C1	D2	W3	X1	M1						M9
						[42]						[41]
						Sample: M1(1)	M1(2)	M1(3)	M1(4)	M1(5)	M1(6)	
SiO_2_	45.18	45.16	46.11	43.89	47.64	39	41	44.9	42.2	43.7	43.3	33.1
TiO_2_	0.54	0.53	0.52	0.53	0.46	0.7	0.6	0.7	0.6	0.7	0.6	0.65
Al_2_O_3_	10.33	8.94	9.75	9.57	10.93	10.7	9.8	10.8	8.5	12.4	10.6	7.51
Fe_2_O_3_	4.52	3.85	4.10	4.20	4.10	3.6	3	3.6	3.2	4.3	3	4.35
MnO	0.09	0.09	0.09	0.09	0.08	-	-	-	-	0.1	-	0.07
MgO	4.34	4.09	4.31	4.17	3.39	5.7	6.6	6.8	6.3	5.6	5.6	5.77
CaO	16.31	18.20	16.61	18.02	15.57	15.7	13.3	12.9	17.8	14.3	14.3	22.07
K_2_O	2.12	1.88	1.91	2.07	2.45	2.7	2.5	2.8	2.4	2.9	2.5	1.84
Na_2_O	1.28	1.33	1.43	1.46	1.31	1.5	2.6	1.4	1	1.3	2	0.53
P_2_O_5_	0.14	0.13	0.14	0.13	0.13	<0.01	<0.01	<0.01	<0.01	<0.01	<0.01	0.14
L.O.I	17.68	17.10	17.35	18.96	16.31	18.3	18.3	15.9	17.8	14.6	17	23.49
Sum	102.54	101.29	99.93	103.09	102.37	97.0	97.7	99.8	99.8	99.9	98.9	99.52

**Table 9:** Trace elements analysed by XRF for adobe and mud–straw samples in this study (concentrations in ppm).

Trace Elements	Adobe Type			Mud–Straw Type	
	A4	C1	D2	W3	X1
Ba	619	749	646	855	485
Co	17	14	15	16	15
Ni	85	69	77	73	80
Cu	45	50	42	132	45
Zn	115	95	98	106	94
Cr	132	119	142	121	119
Sr	558	575	628	590	466
Y	58	60	61	58	74
Zr	218	221	231	213	228
Ga	22	22	24	22	25
Nb	79	86	86	85	106
Ce	75	73	72	70	82

**Table 10:** Mineralogical composition identified in the XRD test for adobe and mud–straw samples in this study and by other researchers.

Compound Name	Chemical Formula	This Study					Other Researchers								
		Location													
		Maybod					Ardakan	Jarquyeh	Yazd	Esfarayen		Susa			
		Adobe			Mud–Straw		Adobe								
		A4	C1	D2	W3	X1	M1	M2	M3	M4	M5	M6	M7	M8	M9
							[42]	[43]		[40]		[41]			
Quartz	SiO_2_	●	●	●	●	●	●	●	●	●		●	●	●	●
Albite	Na(AlSi_3_O_8_)	●	●	●		●					●	●	●		
Albite, ordered	NaAlSi_3_O_8_	●	●	●	●	●		●	●						
Orthoclase	(K_0.94_Na_0.06_)(AlSi_3_O_8_)	●	●	●	●	●									
Calcite	CaCO_3_	●	●	●	●	●	●	●	●	●	●	●	●	●	●
Clinochlore	Mg_5_Al(SiAl)_4_O_10_(OH)_8_	●	●	●	●	●		●	●						●
Dolomite	CaMg(CO_3_)_2_	●	●	●	●	●	●	●	●					●	
Illite	K(Al_4_Si_2_O_9_(OH)_3_)	●	●	●	●	●	●								
Hematite	Fe_2_O_3_	●		●		●									
Palygorskite	Mg_5_(SiAl)_8_O_20_(OH)_2.8_		●	●											
Ferroactinolite	(Ca,Na,K)_2_Fe_5_Si_8_O_22_		●												
Cordierite	Mg_2_Al_4_Si_5_O_18_			●											
Heulandite	(X)_3_(Al_3_Si_9_O_24_)*7-8H_2_O			●											
Hydrotalcite	(Mg_0.667_Al_0.333_)					●									
Feldspar	NaAlSi_3_O_8_—CaAl_2_Si_2_O_8_						●							●	
Kaolinite	Si_2_Al_2_O_5_(OH)_4_						●								
Chlorite	(Fe,Mg,Al)_6_(SiAl)_4_O_10_(OH)_8_						●								
Smectite	(Ca,Na,H)(Al,Mg,Fe,Zn)_2_ (SiAl)_4_O_10_(OH)_2-x_H_2_O						●								
Muscovite	KAl_2_(AlSi_3_O_10_)_3_(F.OH)_2_							●	●	●	●	●	●		
Microcline	KAlSi_3_O_8_								●						
Biotite	K(Mg,Fe)_3_AlSi_3_O_10_(F,OH)_2_									●					
Enstatite	MgFe(Si_2_O_6_)										●		●		
Apatite	Ca_5_(PO_4_)_3_(F,Cl,OH)										●	●	●		
Gypsum	CaSO_4_,2H_2_O					●									●

**Table 11:** Average moisture content of adobe and mud–straw (containing straw) samples in this study for test duration of 30 days.

Material	Code	Location	Period	Straw Content (wt%)	Average Moisture Content at RH = 60% (EMC_60_) (%)	Average Moisture Content at RH = 80% (EMC_80_) (%)	Average Moisture Content at RH = 95% (EMC_95_) (%)	Cl^−^ (wt%)	Na^+^ (wt%)	NaCl at RH = 15% (mol)
Adobe	A4	Narin Castle (Maybod)	2017	0.58	1.83	3.50	7.10	0.13	0.12	2.25 × 10^−4^
	C1	Kazem Abad village (Maybod)	Probably 1870	0	1.10	2.10	4.40	0.07	0.09	1.43 × 10^−4^
	D2	Narin Castle (Maybod)	Probably 1320	0.02	1.63	3.10	6.40	0.14	0.11	1.94 × 10^−4^
Mud–Straw	W3	Deh-Naw Mosque (Maybod)	2019	11	2.47	4.80	15.30	0.81	0.54	11.36 × 10^−4^
	X1	Deh-Naw Mosque (Maybod)	2009	8	1.54	3.10	7.40	0.06	0.07	1.22 × 10^−4^

**Table 12:** Water loss and carbon dioxide loss of completely dry and saturated adobe and mud–straw samples measured by TGA and DSC.

Material	Code	H_2_O Loss (%)		CO_2_ Loss (%)		CaO Analysed by XRF (wt%)
		Completely Dry	Saturated	Completely Dry	Saturated	
Adobe	A4	1.14	2.03	12.41	11.79	15.82
	C1	1.18	2.33	13.91	13.39	17.92
	D2	1.74	3.38	11.86	11.20	16.16
Mud–Straw	W3	1.32	8	12.53	10.74	17.35
	X1	1.38	14.08	12.04	10.04	15.14

## Data Availability

The data that support the findings of this study are available on request from the corresponding author.
